# Analysis of the transcriptomic, metabolomic, and gene regulatory responses to *Puccinia sorghi* in maize

**DOI:** 10.1111/mpp.13040

**Published:** 2021-02-28

**Authors:** Saet‐Byul Kim, Lisa Van den Broeck, Shailesh Karre, Hoseong Choi, Shawn A. Christensen, Guan‐Feng Wang, Yeonhwa Jo, Won Kyong Cho, Peter Balint‐Kurti

**Affiliations:** ^1^ Department of Entomology and Plant Pathology NC State University Raleigh North Carolina USA; ^2^ Department of Plant and Microbial Biology NC State University Raleigh North Carolina USA; ^3^ Research Institute of Agriculture and Life Sciences College of Agriculture and Life Sciences Seoul National University Seoul Republic of Korea; ^4^ Chemistry Research Unit Department of Agriculture–Agricultural Research Service (USDA‐ARS) Center for Medical, Agricultural, and Veterinary Entomology Gainesville Florida USA; ^5^ The Key Laboratory of Plant Development and Environmental Adaptation Biology Ministry of Education School of Life Sciences Shandong University Qingdao China; ^6^ Plant Science Research Unit USDA‐ARS Raleigh North Carolina USA

**Keywords:** common rust, gene regulatory network, maize, *Puccinia sorghi*, RNA‐Seq

## Abstract

Common rust, caused by *Puccinia sorghi*, is a widespread and destructive disease of maize. The *Rp1‐D* gene confers resistance to the *P. sorghi* IN2 isolate, mediating a hypersensitive cell death response (HR). To identify differentially expressed genes (DEGs) and metabolites associated with the compatible (susceptible) interaction and with *Rp1‐D*‐mediated resistance in maize, we performed transcriptomics and targeted metabolome analyses of *P. sorghi* IN2‐infected leaves from the near‐isogenic lines H95 and H95:Rp1‐D, which differed for the presence of *Rp1‐D*. We observed up‐regulation of genes involved in the defence response and secondary metabolism, including the phenylpropanoid, flavonoid, and terpenoid pathways. Metabolome analyses confirmed that intermediates from several transcriptionally up‐regulated pathways accumulated during the defence response. We identified a common response in H95:Rp1‐D and H95 with an additional H95:Rp1‐D‐specific resistance response observed at early time points at both transcriptional and metabolic levels. To better understand the mechanisms underlying *Rp1‐D*‐mediated resistance, we inferred gene regulatory networks occurring in response to *P. sorghi* infection. A number of transcription factors including WRKY53, BHLH124, NKD1, BZIP84, and MYB100 were identified as potentially important signalling hubs in the resistance‐specific response. Overall, this study provides a novel and multifaceted understanding of the maize susceptible and resistance‐specific responses to *P. sorghi*.

## Graphical abstract

We document the consequences of maize common rust infection of near‐isogenic resistant (a) and susceptible (b) maize lines at the transcription (c), metabolomic (d), and gene network (e) levels.

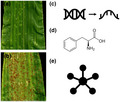

## 1 INTRODUCTION

The plant innate immune system is often represented as comprising two tiers: pathogen‐associated molecular pattern (PAMP)‐triggered immunity (PTI) and effector‐triggered immunity (ETI) (Bent & Mackey, 2007). PTI is a basal defence response activated by the recognition of PAMPs, usually conserved molecules with general microbial functions, via pattern recognition receptors (PRRs) at the cell surface. ETI is a more specific defence response triggered by the recognition of pathogen‐secreted proteins that facilitate the pathogenesis process, called effectors, via intracellular immune receptors called R proteins. Most R proteins carry nucleotide‐binding sites (NBS) and leucine‐rich repeat (LRR) domains, known as NLRs (Jones & Dangl, 2006). Each NLR R protein recognizes the presence of a specific effector, triggering a multifaceted defence response often including a rapid localized cell death at the point of pathogen penetration called the hypersensitive response (HR), generation of reactive oxygen species (ROS), induction of defence‐related gene expression, and production of secondary metabolites and phytohormones (Balint‐Kurti, 2019). Several authors have proposed alternatives to this ETI/PTI paradigm (e.g., Cook et al., 2015) based on the observation that the distinctions between PRRs and R proteins and between PAMPs and effectors are often not clearcut (Thomma et al., 2011). While this is true, the ETI/PTI paradigm serves as a convenient classification system for most plant immune receptor‐based responses and will be used here.

The defence response also often includes the synthesis of several secondary metabolites including a number of phenylpropanoids and terpenoids (Kushalappa et al., 2016). The phenylpropanoid pathway (PPP), which starts with the conversion of phenylalanine to *trans*‐cinnamic acid by the enzyme phenylalanine ammonia‐lyase (PAL), has long been recognized for its importance in disease resistance and the defence response in higher plants (Dixon et al., 2002; Naoumkina et al., 2010; Vogt, 2010). The conversion of the PPP intermediate 4‐coumaroyl‐CoA to naringenin chalcone by the polyketide synthase enzyme chalcone synthase constitutes the first committed step in the flavonoid biosynthesis pathway (Winkel‐Shirley, 2002). Products of the PPP are also channelled for lignin biosynthesis by the activity of two enzymes, cinnamoyl CoA reductase and cinnamoyl alcohol dehydrogenase, which catalyses the reduction of Co‐A thioesters (Vogt, 2010). Another significant PPP subsidiary pathway involves biosynthesis of hydroxycinnamic acid amides (HCAAs), which are polymers made of hydroxycinnamic acids and polyamines, catalysed by amine‐specific hydroxycinnamoyl transferases (Facchini et al., 2002). Phenylpropanoids, HCAAs, and lignins restrict pathogen advancement through cell wall thickening and flavonoids/flavonoid glucosides act as signalling molecules, quench ROS, and display antimicrobial properties against invading pathogens (Dixon et al., 2002).

Terpenoids probably comprise the largest class of plant secondary metabolites (Yazaki et al., 2017) and are known to play a major role in the responses to biotic and abiotic stress (Schmelz et al., 2011; Singh & Sharma, 2015). Terpenoid phytoalexins called kauralexins accumulate in maize in response to infection with both *Cercospora zeina*, the causal agent of grey leaf spot (Meyer et al., 2017), and *Fusarium graminearum*, the causal agent of Gibberella ear and stalk rot (Huffaker et al., 2011). Kauralexin B3 reduces the in vitro growth of the opportunistic fungal necrotroph *Rhizopus microsporus* and the causal agent of anthracnose stalk rot, *Colletotrichum graminicola* (Schmelz et al., 2011).

Within maize, the majority (c.70%) of genome‐wide association study (GWAS) hits fall outside of annotated genes in intergenic regions, emphasizing the importance of *cis*‐regulatory mechanisms (Wallace et al., 2014). The expression of enzyme‐encoding genes needed for the biosynthesis of secondary metabolites is regulated by transcription factors (TFs) that bind to *cis*‐regulatory elements in the promoter region (Yang et al., 2017). These TFs and their downstream target genes form interconnected gene regulatory networks (GRNs) that regulate a variety of biological processes and environmental responses. In maize, using yeast one‐hybrid assays, a GRN that underlies the transcriptional control of genes involved in the biosynthesis of secondary metabolites was generated (Gomez‐Cano et al., 2020; Yang et al., 2017). These studies identified 1,100 regulatory interactions between 54 phenolic gene promoters and 568 TFs and identified 11 key TFs that regulated 10 or more genes. GRNs can also be constructed with computational inference methods, most of them levering gene expression data (Spurney et al., 2020). Genome‐wide expression profiling and network inference with targeted metabolite analysis is a comprehensive and innovative approach to identify key upstream TFs that regulate secondary metabolite accumulation, important for plant interactions with the environment (Zhou et al., 2020).

Species of the fungal genus *Puccinia* cause rust diseases and are among the most devastating groups of crop pathogens (Aime et al., 2017). The obligate biotrophic fungus *Puccinia sorghi*, causal agent of common rust disease of maize, is responsible for significant reductions in maize yield in North and South America and in other temperate maize‐growing regions worldwide (Darino et al., 2016; Pataky, 1987; Ramirez‐Cabral et al., 2017; Shah & Dillard, 2006).

The maize *Rp1* locus on the short arm of chromosome 10 carries variable numbers of tandemly repeated NLR genes (Hulbert, 1997). Race‐specific resistance to common rust was reported to be controlled by at least 14 genes at this locus, designated *Rp1‐A* to *Rp1‐N* (Saxena & Hooker, 1968). Subsequently it was reported that some of these specificities are probably closely linked rather than allelic (Hooker, 1985; Hulbert & Bennetzen, 1991). *Rp1‐D* was identified as a gene encoding a coiled coil (CC)‐NLR protein conferring HR‐mediated resistance to *P. sorghi* isolate IN2 (Collins et al., 1999). Macroscopic HR conferred by *Rp1‐D* was observed at 24 hr postinoculation (hpi) with no rust spores being detectable on infected leaves, while spore formation was apparent about 6 days postinoculation (dpi) in a near‐isogenic maize line lacking *Rp1‐D* (Morris et al., 1998).

Due to its complex, repetitive nature, the *Rp1* locus is highly unstable and recombinogenic (Ayliffe et al., 2004; Hulbert & Bennetzen, 1991; Richter et al., 1995; Smith & Hulbert, 2005; Smith et al., 2010; Sudupak et al., 1993). *Rp1‐D21* is a chimeric gene derived from intragenic recombination between two paralogs, *Rp1‐dp2* and *Rp1‐D*. It carries the CC and NBS domains and the N‐terminal third of the LRR domain of Rp1‐dp2 and the rest of the LRR domain from Rp1‐D (Smith et al., 2010; Sun et al., 2001). Rp1‐D21 is autoactive and causes spontaneous HR in maize and tobacco in the absence of pathogens (Chintamanani et al., 2010; Smith et al., 2010; Wang, Ji, et al., 2015). Wang and Balint‐Kurti (2016) showed that two PPP enzymes important for lignin biosynthesis, hydroxycinnamoyl transferase (HCT) and caffeoyl CoA *O*‐methyltransferase (CCoAOMT), play a role in regulating Rp1‐D21‐induced HR, probably via physical interaction.

In this study, we explored genome‐wide expression analyses and targeted metabolome analyses upon *P. sorghi* infection in the near‐isogenic H95:Rp1‐D (resistant) and H95 (susceptible) lines. After confirming the near‐identical genetic makeup between H95:Rp1‐D and H95, we proceeded to unravel some of the regulatory and metabolic interactions associated with the defence response and with resistance in the H95:Rp1‐D line. Using targeted metabolomics, we found a strong induction of the PPP as well as the phytoalexin pathway upon infection in both lines, coinciding with increased expression of several genes encoding enzymes that catalyse these metabolic pathways. Additionally, several PPP compounds, including 4‐coumaric acid and naringenin, specifically accumulated in the H95:Rp1‐D resistant line. Through GRN inference, we identified regulatory interactions and key TFs, such as WRKY53 and MYB100, that may underlie Rp1‐D resistance. These data provide a detailed understanding of the maize compatible and Rp1‐D‐mediated incompatible transcriptional and metabolomic responses to infection with *P. sorghi* and their underlying GRNs.

## 2 RESULTS

### 2.1 Near‐isogenic lines to decipher fungal immune responses

To avoid the confounding effects of genetic background, we used the near‐isogenic lines H95 and H95:Rp1‐D, which have been reported to differ just for *Rp1‐D* (Pataky, 1987; Smith et al., 2010). These two lines appeared phenotypically very similar when grown in the field or greenhouse and had been reported previously to be near‐isogenic, but their exact derivation and relationship was unclear. To confirm that they were near‐isogenic, both lines, as well as the commonly used maize lines B73 and Mo17, were compared using the random amplified polymorphic DNA (RAPD) molecular fingerprinting technique (Williams et al., 1990). RAPD reactions performed using a set of seven 10‐base primers (Table S1) showed that H95:Rp1‐D and H95 produced very similar patterns on agarose gels, indicating that they were highly genetically similar, and as expected, were distinct from B73 and Mo17 (Figure S1). The subsequent global expression analyses we performed (see below) confirmed this close relationship. We observed only 19 differentially expressed genes (DEGs) between H95 and H95:Rp1‐D prior to infection (File S1). Of these DEGs, four mapped to approx. 1.5 to 4 Mb on chromosome 10, where the *Rp1* locus itself is situated, indicating that these are likely non‐H95 alleles that have been introduced into H95:Rp1‐D by linkage drag with *Rp1‐D* itself. Three other chromosomal regions carried more than one of these DEGs (Chr. 2 c.196.38–199.21 Mb, Chr. 6 37.47–37.48 Mb, and Chr. 8 c.177.0–178.7 Mb), indicating that these regions probably represent three other small introgressions that differentiate H95 from H95:Rp1‐D. Twelve of these 19 genes mapped to four loci comprising less than 10 Mb, less than 0.5% of the genome. It is clear that H95 and H95:Rp1‐D are highly isogenic and that the observed and quantified differences between the susceptible H95 and resistant H95:Rp1‐D lines can largely be ascribed to activation of *Rp1‐D* and to the plant’s immune response.

### 2.2 Avirulent *P. sorghi* IN2 induces HR cell death in H95:Rp1‐D

**FIGURE 1:**
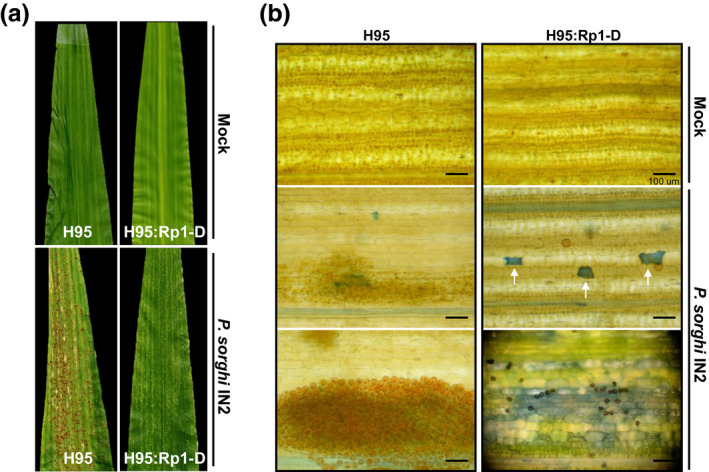
Comparison of leaves of H95 (susceptible) and H95:Rp1‐D (resistant) lines infected with *Puccinia sorghi* IN2. Fourteen‐day‐old seedlings were infected and leaf materials were imaged at 7 days postinoculation (dpi). (a) Macroscopic images showing sporulation on infected H95 leaves while necrotic flecks but no infection is apparent on infected H95:Rp1‐D leaves. No infection or necrotic flecks are observed on the mock‐inoculated controls. (b) Microscopic images of infected H95 and H95:Rp1‐D leaves. Dead leaf cells are stained by trypan blue and fungal spores are visible as brown circles. Infected H95:Rp1‐D leaves display both patches of cell death and single dead cells caused by the hypersensitive response. H95 leaves display abundant fungal sporulation. Scale bar represents 100 µm

Initially, we compared the phenotypic immune response to *P*. *sorghi* between the H95 and near‐isogenic H95:Rp1‐D lines. We inoculated 14‐day‐old H95:Rp1‐D and H95 maize seedlings with *P. sorghi* IN2, which is avirulent to lines carrying *Rp1‐D*, and evaluated the phenotype at 7 dpi. HR cell death was observed on H95:Rp1‐D, while abundant sporulation was apparent on H95 at 7 dpi (Figure 1a). Trypan blue staining of dead cells indicated that HR cell death on the H95:Rp1‐D leaves occurred both at the single‐cell level and as patches of dead cells. No individual dead cells were observed on mock‐inoculated leaves of either genotype (Figure 1b). This confirms that the *P. sorghi* IN2 isolate can trigger HR and ETI in H95:Rp1‐D in an Rp1‐D‐dependent manner.

### 2.3 *P. sorghi* infection induces a resistance‐specific response in H95:Rp1‐D

To explore the transcriptome changes occurring in response to *P. sorghi* infection, infected leaves from both H95:Rp1‐D and H95 lines and mock‐inoculated H95 plants were sampled at 0, 12, 24, and 120 hpi. These time points corresponded to specific stages of the incompatible response: immediately prior to infection (0 hpi), before HR was observable (12 hpi), when the HR was first observable (24 hpi), and after HR lesions had stopped expanding in the incompatible interaction and when spores were first apparent in the compatible interaction (120 hpi). After RNA sequencing (RNA‐Seq), reads were mapped to v4 of the B73 reference genome and the *P. sorghi* RO10H11247 genome (Rochi et al., 2018). The number of reads obtained per sample ranged from 42 to 253 million, of which, except for the 120 hpi time point in H95 samples, 69%–87% mapped to the B73 reference genome (File S2) and 0.08%–1.62% mapped to the *P. sorghi* reference genome (File S2). At 120 hpi, the H95 samples had a remarkably high proportion of reads mapping to *P. sorghi* (39%–44%) and the lowest proportion to B73 (32%–37%). To explore variation within the RNA‐Seq data set, we performed a principal component analysis (PCA) and evaluated the replicates of all samples based on the similarity of gene expression profiles. The three replicates of each sample clustered together based on their first and second PC, demonstrating the robustness of the data set (Figure S2). Most of the mock samples and H95 and H95:Rp1‐D samples prior to infection grouped together as expected. Interestingly, the global expression profiles of H95:Rp1‐D at 120 hpi are more similar to uninfected than infected samples, suggesting that the response to *P. sorghi* in H95:Rp1‐D (but not in H95) had finished by 120 hpi.

A quantitative reverse transcription PCR (RT‐qPCR) was performed on a set of six DEGs that are involved in the defence responses, as a spot‐check on the RNA‐Seq data. Overall, as expected, levels and patterns of gene induction determined by RT‐qPCR were similar to those determined by RNA‐Seq (Figure S3).

**FIGURE 2:**
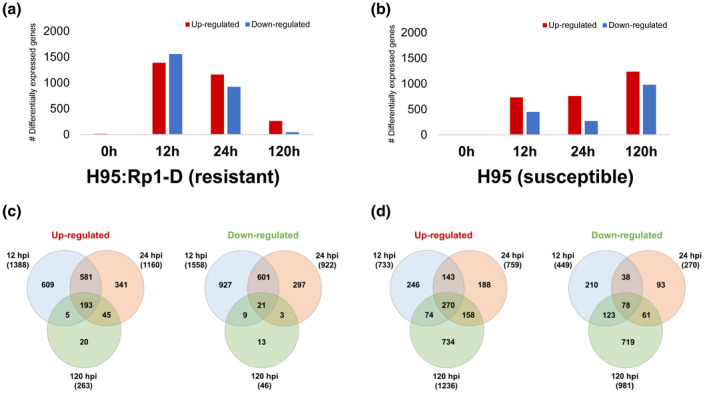
Number of differentially expressed genes (DEGs) identified in (a) H95:Rp1‐D and (b) H95 infected with *Puccinia sorghi* *IN2* at 12, 24, and 120 hr postinoculation (hpi), respectively. (c, d) Venn diagram indicating the distribution of DEGs among time points in (c) H95:Rp1‐D and (d) H95

We observed a strong transcriptional response in both lines but, using the number of DEGs as the criterion, the H95:Rp1‐D response was stronger and faster than the response of the susceptible H95 line (Figure 2a,b). Lower numbers of DEGs were detected in H95 at 12 hpi compared to H95:Rp1‐D, while at 120 hpi the opposite was observed: an increased transcriptional response in H95 and a lower number of DEGs in H95:Rp1‐D. This is in line with the PCA results described above (Figure 2a,b). There were a limited number of DEGs common to all time points (Figure 2c,d), suggesting the different response states through which the host progresses during the course of infection. For 12 and 24 hpi, 95.6% and 89.0%, respectively, of the DEGs identified in H95 were also found among the H95:Rp1‐D DEGs (Figure S4). In addition to this common response between H95 and H95:Rp1‐D to the infection, H95:Rp1‐D also appeared to generate a unique response to *P. sorghi* with more than 50% of the DEGs in H95:Rp1‐D at 12 and 24 hpi not found in the equivalent H95 time points. (Figure S4). We should note that 30% and 39% of the genes in these resistance‐specific 12 and 24 hpi DEG sets, respectively, were differentially expressed at 120 hpi in the susceptible H95 line. This suggests that one important component of the unique response is in the timing of induction.

### 2.4 Phenylpropanoid and terpenoid biosynthetic genes are strongly regulated in response to *P. sorghi* infection

**FIGURE 3:**
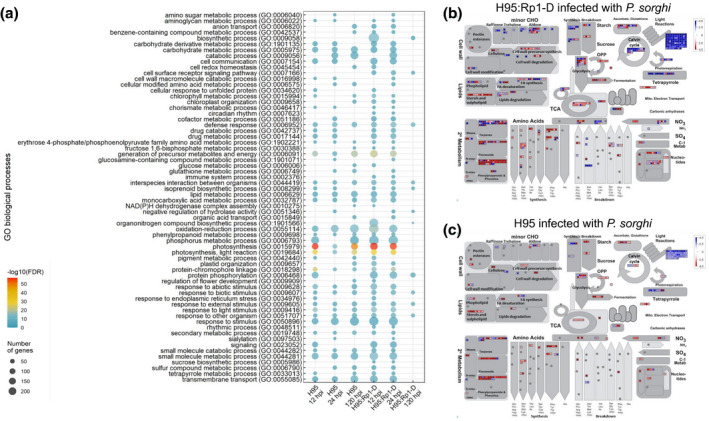
(a) Overview of the distribution of differentially expressed genes (DEGs) from *Puccinia sorghi* IN2‐infected H95:Rp1‐D and H95 plants. (a) Selected Gene Ontology (GO) terms that are enriched among DEGs from infected H95:Rp1‐D and H95 plants at 12, 24, and 120 hr postinoculation (hpi). The GO terms in each biological process category are listed along the *y* axis. The horizontal axis shows the hosts and time points. (b, c) The distribution of DEGs from infected H95:Rp1‐D and H95 plants at 12 hpi among various cellular processes, visualized by MapMan. The intensity of the colour indicates the level of differential expression. Scale bar displays log_2_(fold change) values. Red and blue colours represent up‐ and down‐regulation, respectively

To further characterize the response to *P. sorghi* in the susceptible H95 and resistant H95:Rp1‐D lines, we performed Gene Ontology (GO) enrichment and MapMan analyses. GO enrichment analysis identified 230 enriched processes among the up‐regulated DEGs for both resistant and susceptible lines (File S3). Enriched GO categories included oxidation‐reduction process, photosynthesis, secondary metabolite, and defence response genes (Figure 3a). Over the full set of enriched GO terms, there were 188 and 157 different GO terms enriched among the up‐regulated DEGs from H95:Rp1‐D and H95, respectively. Of these, 115 (50%) of the terms were shared between the two genotypes (File S3), indicating a common response between H95 and H95:Rp1‐D, as might be expected from the proportion of shared DEGs at these time points discussed above (Figure S4).

To visualize the likely effects of the DEGs in the H95:Rp1‐D and H95 interactions at each time point, MapMan analysis was performed. This analysis identified similar pathways to the GO enrichment analysis for all three time points in both the susceptible and resistant lines (Figures 3b,c and S5). This is in line with the RNA‐Seq analysis where c.90% of the DEGs in the susceptible line overlap with the DEGs of the resistant line. Most highly induced DEGs were associated with secondary metabolism including “Phenylpropanoids & Phenolics” and “Terpenes,” and most down‐regulated DEGs were associated with the light reaction (corresponding to enriched GO terms in the photosynthesis category).

### 2.5 Secondary metabolites are strongly induced in maize upon *P. sorghi* infection

**FIGURE 4:**
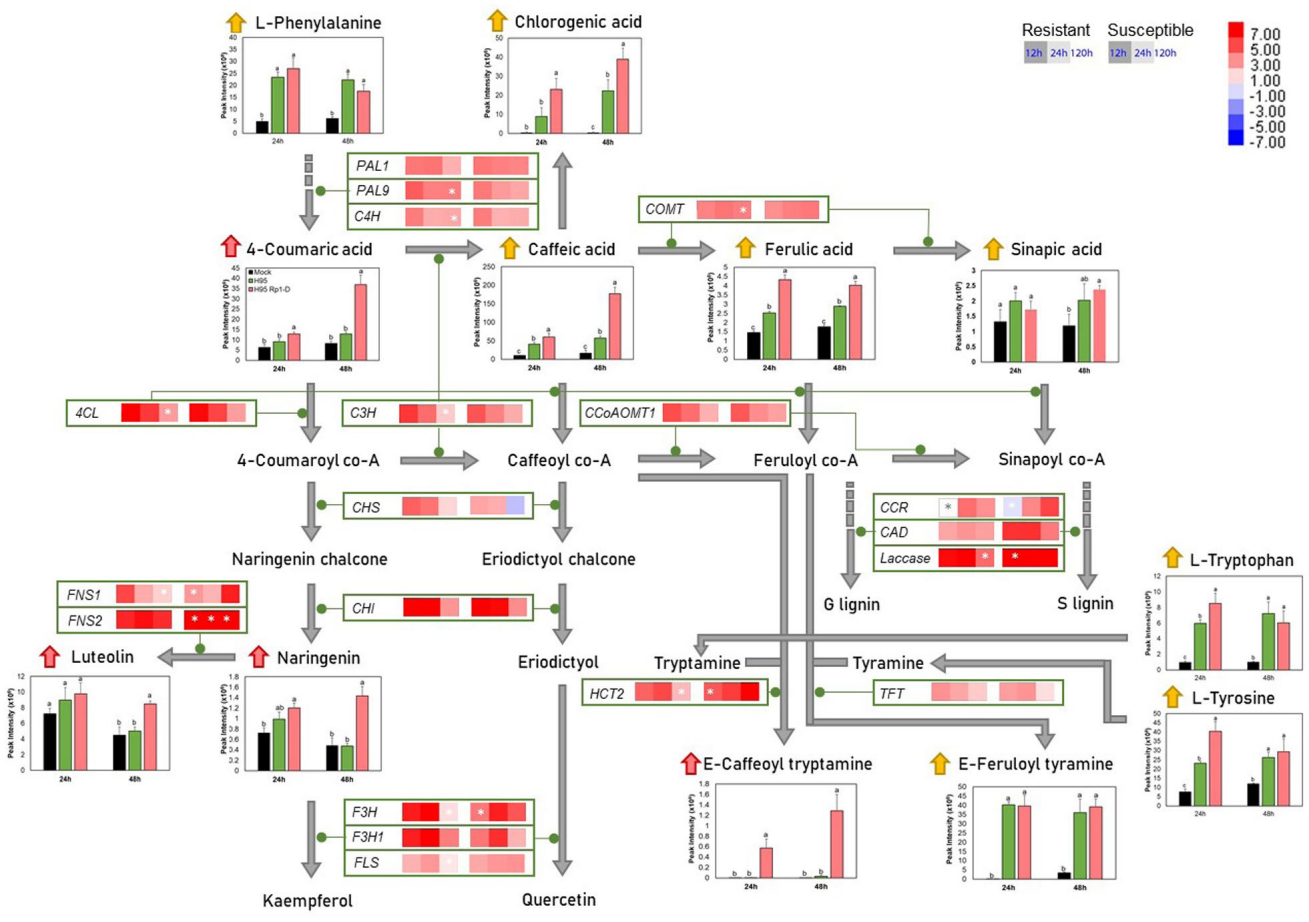
A schematic of the phenylpropanoid and associated pathways showing selected differentially expressed enzyme‐encoding genes in H95 (susceptible) or H95:Rp1‐D (resistant) lines 12, 24, and 120 hr postinoculation (hpi) with *Puccinia sorghi* IN2 and the relative levels of specific metabolites in H95 (green bar) and H95:Rp1‐D (pink bar) plants infected with *P. sorghi* IN2 or mock‐inoculated plants (black bar) 24 and 48 hpi. Metabolite peak intensities (*n* = 4, average ± *SEM*) are depicted on the primary *y* axis. Letters (a–c) represent significantly different treatments (*p* < .05, one‐way analysis of variance comparison followed by Duncan's test). The vertical scale bar in the top left shows the colours assigned to levels of differential expression (log_2_[fold change] values) compared to mock. Pink arrows represent accumulated metabolites in the H95:Rp1‐D line, while yellow arrows represent accumulated metabolites in both H95 and H95:Rp1‐D lines. Green lines indicate the stages in the metabolic pathways the protein encoded by each gene is thought to catalyse. Asterisks indicate no significant change (*p* > .05)

**FIGURE 5:**
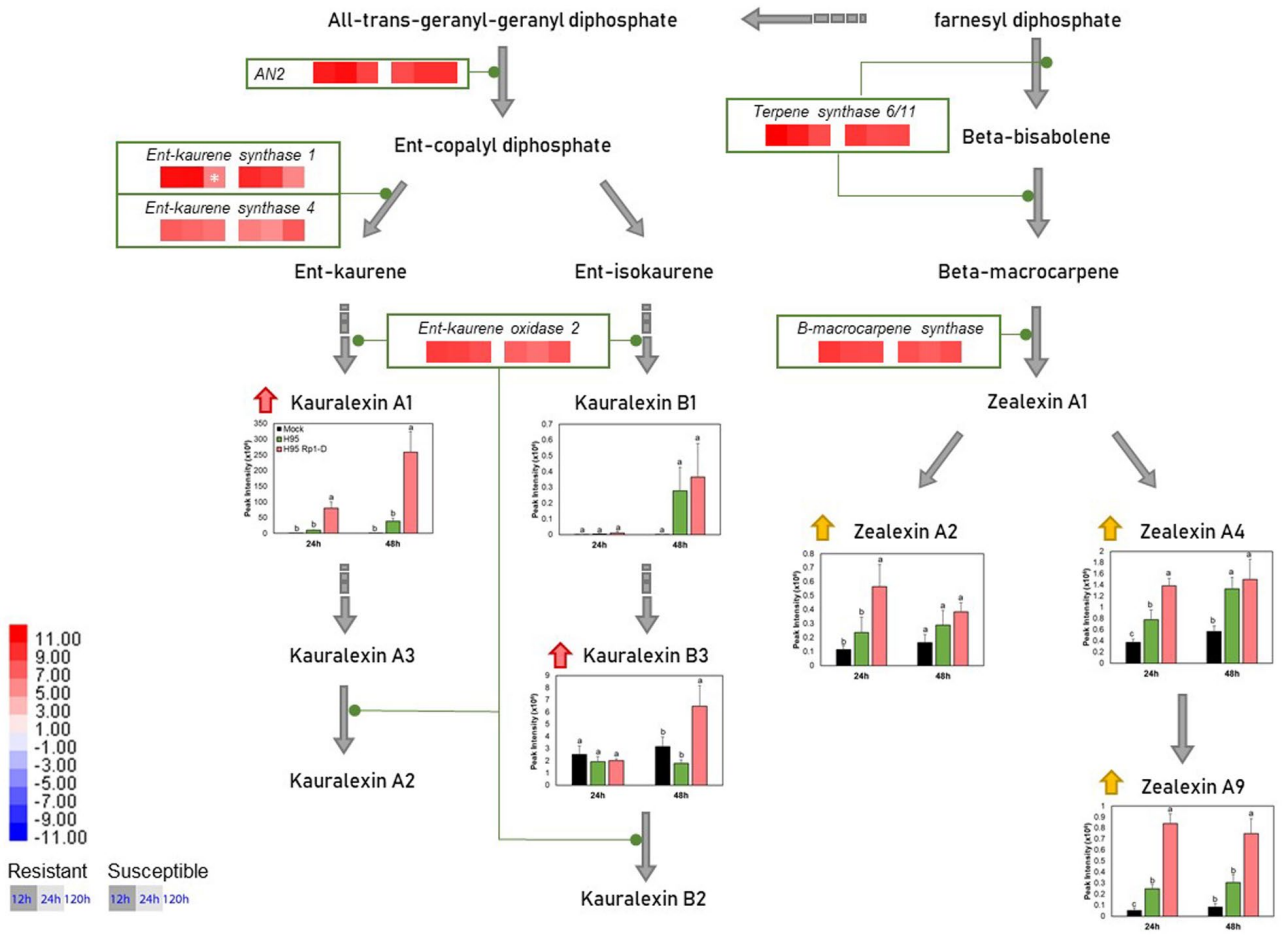
A schematic of the maize terpenoid pathway showing differentially expressed enzyme‐encoding genes in the H95 (susceptible) and H95:Rp1‐D (resistant) lines 12, 24, and 120 hr postinoculation (hpi) infected with *Puccinia sorghi* IN2 and the relative levels of accumulation of specific metabolites in H95 (green bar) and H95:Rp1‐D (pink bar) plants infected with *P. sorghi* IN2 or mock‐inoculated plants (black bar) 24 and 48 hpi. Metabolite peak intensities (*n* = 4, average ± *SEM*) are depicted on the primary *y* axis. Letters (a–c) represent significantly different treatments (*p* < .05, one‐way analysis of variance comparison followed by Duncan's test). The vertical scale bar in the top left shows the colours assigned to levels of differential expression (log_2_[fold change] values) compared to mock. Pink arrows represent accumulated metabolites in the H95:Rp1‐D line, while yellow arrows represent accumulated metabolites in both H95 and H95:Rp1‐D lines. Green lines indicate the stages in the metabolic pathways the protein encoded by each gene is thought to catalyse. Asterisks indicate no significant change (*p* > .05)

We used targeted metabolomics to investigate whether the metabolic pathways predicted from our analyses to be associated with the common and the resistance‐specific defence responses were modulated as predicted. Infected H95 and H95:Rp1‐D plants and mock‐inoculated H95 plants were harvested at two time points based on the development of Rp1‐D‐mediated HR in the resistant line: visual emergence of HR (24 hpi) and stabilization of HR (48 hpi). We focused on four pathways identified by the GO enrichment analysis: the PPP, two of its offshoot pathways (the HCAA pathway and the flavonoid pathway), and the terpenoid biosynthetic pathway. Figures 4 and 5 illustrate the differential expression we observed for genes encoding enzymes in these pathways, the positions of the encoded enzymes in the metabolic pathways, and the levels of various metabolite intermediates.

The PPP serves as a starting point for the production of many other important metabolites, such as the flavonoids and coumarins, and is required for the biosynthesis of lignin. The levels of PPP intermediates caffeic acid, ferulic acid, and chlorogenic acid were significantly increased at 24 and 48 hpi. The degree of induction of all three metabolites was higher in the H95:Rp1‐D resistant line compared to H95 at both time points (Figure 4). All phenylpropanoids are derived from phenylalanine, which itself was significantly increased in the resistant interactions. Interestingly, 4‐coumaric acid, the direct derivative of phenylalanine in the PPP, was significantly induced in the resistant but not in the susceptible line (Figure 4). 4‐Coumaric acid is also the starting point for the biosynthesis of naringenin, an antimicrobial compound, which can further be converted to luteolin in the flavonoid pathway. Both naringenin and luteolin were also specifically induced in the resistant line (Figure 4). In most, but not all cases, PPP and flavonoid biosynthetic genes were induced at higher levels in H95:Rp1‐D at 12 and 24 than at 120 hpi (Figure 4). Two other compounds from the PPP, sinapic acid and *E*‐caffeoyl tryptamine, were increased in the resistant H95:Rp1‐D line upon *P. sorghi* infection at 48 hpi (Figure 4). Sinapic acid, caffeic acid, and ferulic acid are all input compounds for biosynthesis of lignin building blocks. Laccase, an enzyme that catalyses the conversion of these compounds to lignins, was highly induced upon infection within the resistant and susceptible lines. The gene encoding tryptamine HCT2 (*Zm00001d030540*), which catalyses the biosynthesis of *E*‐caffeoyl tryptamine, coumaroyl tryptamine, and feruloyl tryptamine, was highly induced in both resistant and susceptible responses at 12 and 24 hpi (Figure 4). Tyramine *N*‐feruloyl transferase (*Zm00001d048407*) catalyses the conversion of tyramine to *E*‐feruloyl tyramine, a compound accumulated in both the resistant and susceptible lines, indicating that this compound might be involved in common defence responses against *P. sorghi*.

Up‐regulation of several diterpenoid biosynthetic genes, including *anther ear 2* (*An2*, *Zm00001d029648*), *ent‐kaurene synthase 1* and *4* (*Zm00001d032858* and *Zm00001d041082*), and *ent‐kaurene oxidase* 2 (*Zm00001d046342*), in both resistant and susceptible responses upon *P. sorghi* infection was observed at 12 and 24 hpi (Figure 5). The products of these enzymes, kauralexin A1 and kauralexin B3, significantly accumulated in H95:Rp1‐D at 48 hpi but not in H95, while the level of kauralexin B1 was increased in both lines at 48 hpi (Figure 5). The levels of three zealexins were increased in both lines but, again, all showed higher accumulation in H95:Rp1‐D (Figure 5).

### 2.6 Salicylic acid is induced in maize under *P. sorghi* infection

**FIGURE 6:**
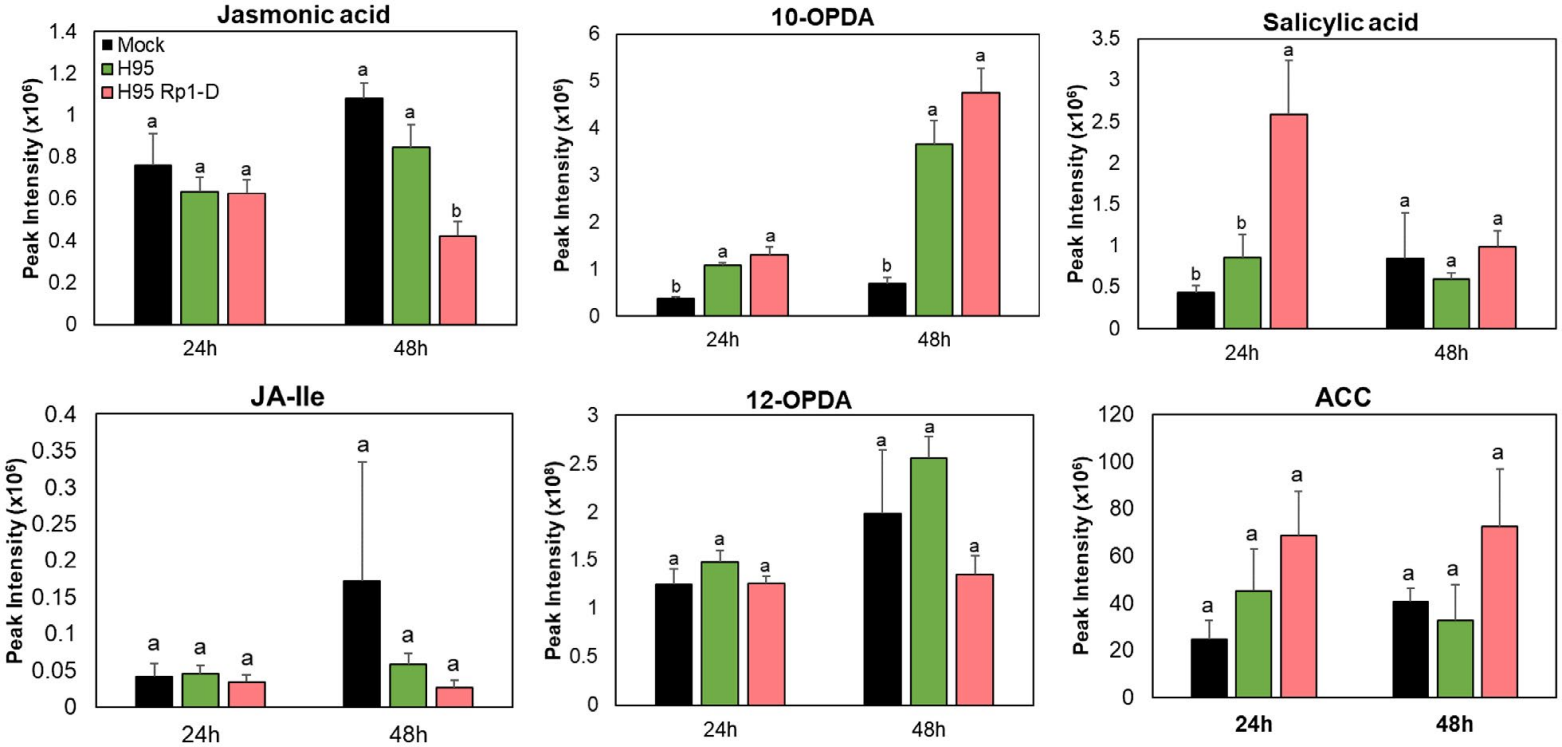
Accumulation of metabolites associated with defence hormone pathways in H95 (susceptible) and H95:Rp1‐D (resistant) lines infected with *Puccinia sorghi* IN2 at 24 and 48 hr postinoculation (hpi). Metabolite peak intensities (*n* = 4, average ± *SEM*) are depicted on the primary *y* axis. Letters (a–c) represent significantly different treatments (*p* < .05, one‐way analysis of variance followed by Duncan's test)

We measured changes in two plant hormones (jasmonic acid [JA] and salicylic acid [SA]), as well as four other compounds (12‐oxo‐phytodienoic acid [12‐OPDA], JA‐Ile, and 10‐oxo‐11‐phytodienoic acid [10‐OPDA] in the oxylipin/JA pathway and 1‐aminocyclopropane‐1‐carboxylic acid [ACC], the direct precursor of the hormone ethylene) (Figure 6). The production of ACC by ACC synthase is the rate‐limiting step in the ethylene biosynthetic pathway (Argueso et al., 2007). Levels of ACC were not altered upon infection in both lines. JA levels were also not altered at 24 and 48 hpi in H95; however, JA showed reduced levels in H95:Rp1‐D at 48 hpi, which is after HR occurred. The levels of JA‐Ile and 12‐OPDA were similar in both lines at all time points. However, the level of 10‐OPDA, which is involved in the “death acid” biosynthetic pathway (Christensen et al., 2015), was moderately induced within 24 hpi, and more highly (>fourfold) in both lines at 48 hpi.

The level of SA was significantly increased in H95:Rp1‐D at 24 hpi but the induction was transient and at 48 hpi the levels of SA in the mock, H95, and H95:Rp1‐D lines were similar (Figure 6). These data suggest that HR induced by *P. sorghi* infection leads to rapid and transient accumulation of SA in H95:Rp1‐D at an early time point.

### 2.7 Gene regulatory network analysis in response to *P. sorghi* infection

**FIGURE 7:**
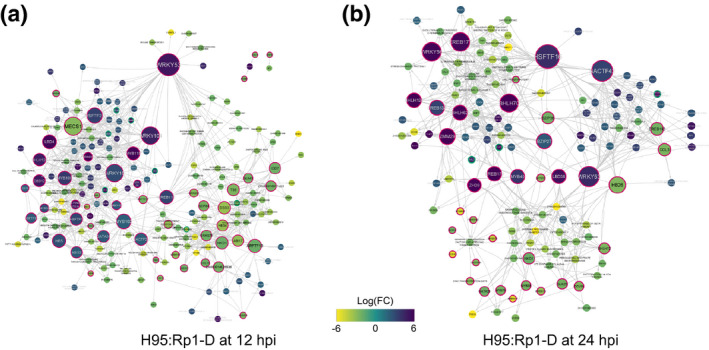
Inferred gene regulatory networks (GRNs) underlying the resistance‐specific response. Causal regulatory interactions were inferred between the differentially expressed genes (DEGs) unique for the H95:Rp1‐D line at 12 hr postinoculation (hpi) (a) and 24 hpi (b). Transcription factors (TFs) and the metabolite‐associated genes have red and green borders, respectively. Each gene was coloured according to induction

As discussed above, in addition to the common defence response upon *P. sorghi* infection, the resistant H95:Rp1‐D line also displayed a unique transcriptional response. We hypothesized that the regulatory mechanisms conferring resistance present in H95:Rp1‐D would probably be reflected in the DEG set unique to H95:Rp1‐D (Figure S4). To further explore this unique DEG set, we inferred the causal regulatory interactions between the TFs and downstream targets in the DEG set unique to H95:Rp1‐D at 12 and 24 hpi using a regression tree algorithm and constructed a GRN for each time point. In total, we predicted the regulatory interactions involving 1,816 DEGs at 12 hpi and 1,169 DEGs at 24 hpi (Figure 7).

Predicted resistance‐specific regulatory proteins belong to several TF families known to be involved in the defence response in other systems, such as WRKY, EREB, and bHLH TFs. Most DEGs were specific for each time point; however, four TFs within the GRN, WRKY53 (*Zm00001d020492*), BHLH124 (*Zm00001d037749*), naked endosperm 1 (NKD1, *Zm00001d002654*), and BZIP84 (*Zm00001d053988*), appeared to play an important role at both time points. To shed light on the processes regulated by the predicted transcriptional interactions, we performed a GO enrichment analysis on the downstream target genes within the GRNs. At 12 hr after infection, GO terms associated with a number of biosynthetic and metabolic processes and responses to a number of biotic and abiotic stimuli were enriched among the downstream target genes (adjusted *p* values < .05; File S5). Within our 12 hpi GRN, a *Laccase* gene (*Zm00001d023617*) was predicted to be regulated by several upstream TFs, including Orphan 175 (*Zm00001d025957*), LBD4 (*Zm00001d028721*), and WRKY104 (*Zm00001d020495*), three TFs noted to regulate genes in the PPP (Yang et al., 2017). We also found the gene encoding flavonoid 3‐monooxygenase (*Zm00001d005823*), involved in flavonol and luteolin biosynthesis in the PPP, was predicted to be activated by three WRKYs, including WRKY104, and two MYB TFs, including MYB118 (*Zm00001d041580*). In addition to MYB118 and the aforementioned TFs, three additional TFs from the 12 hpi GRN, ARF13 (*Zm00001d049295*), MYB100 (*Zm00001d030644*), and Orphan 46 (*Zm00001d004102*), were predicted to regulate metabolic pathway‐associated genes and have previously been shown to be involved in the regulation of secondary metabolites (Yang et al., 2017). In the 24 hpi GRN, MYB40 (*Zm00001d040621*) and WRKY56 (*Zm00001d039532*), both shown to regulate genes in the maize phenolic pathway (Yang et al., 2017), were predicted to regulate PAL7 and naringenin2‐oxoglutarate 3‐dioxygenase, respectively. Analysis of the DEGs in the 24 hpi resistance‐specific GRN identified enriched GO terms associated with secondary metabolic and defence responses, such as response to stimulus, transmembrane transport, phenylpropanoid catabolic process, lignin catabolic process, and photosynthesis (adjusted *p* values < .05; File S5).

To gain insights into the common defence response shared between the susceptible H95 and resistant H95:Rp1‐D, we inferred GRNs from the DEGs common between these two lines and at 12 and 24 hpi (Figures S6 and S7). Interestingly, in several cases, we found that eight and five metabolic‐related genes were predicted to be coregulated by several TFs, including WRKYs, a bHLH, an NAC, and an EREB.

A recent study used chromatin immunoprecipitation (ChIP)‐Seq in protoplasts derived from maize seedlings to examine the physical interactions of a set of 104 maize TFs with their target chromatin sites (Tu et al., 2020). Of these 104 TFs, 23 were also present in the *P. sorghi* response GRNs. The Tu et al. (2020) study identified 40 of the 293 TF–chromatin interactions predicted by our GRNs among these 23 TFs (File S4).

### 2.8 Comparison of maize DEGs induced by activation of Rp1‐D compared to its autoactive derivative Rp1‐D21

*Rp1‐D21* is an autoactive, temperature‐sensitive derivative of *Rp1‐D* that confers a spontaneous HR (Negeri et al., 2013). We had previously conducted an RNA‐Seq analysis identifying DEGs between near‐isogenic B73 × H95 F_1_ plants that differed for the presence of *Rp1‐D21* in the heterozygous condition (Olukolu et al., 2014; Wang, He, et al., 2015). We conducted this analysis in two ways. First, we identified DEGs from 18‐day‐old seedlings maintained under 12‐hr day/12‐hr night conditions in a growth chamber at 22 °C, at which temperature Rp1‐D21 is active (Negeri et al., 2013), here called “Rp1‐D21_constant”. Second, we maintained the seedlings at 30 °C for 14 days, at which temperature Rp1‐D21 is inactive (Negeri et al., 2013), before dropping the temperature to 22 °C for 48 hr, activating the Rp1‐D21 protein, here called “Rp1‐D21_48hr”. In each case the plants were at the five‐leaf stage, and the fourth leaf of the plants was harvested.

The conditions differed substantially between the current study and our previous work in terms of the genotype used, the growth conditions and time of sampling. Nevertheless, we were interested to determine if there were commonalities between the genes induced by normal activation of Rp1‐D due to pathogen detection and by spontaneous activation of the protein encoded by the autoactive allele *Rp1‐D21*. We compared the DEGs identified during Rp1‐D activation at 12 and 24 hpi and in Rp1‐D21_constant and Rp1‐D21_48hr. Not surprisingly, the most distinct of these four conditions was the Rp1‐D21_constant. In this case, HR and the defence response had been constitutively activated since the seedling emerged (probably c.10 days). In total, 1,364 of 2,216 DEGs (62%) up‐regulated in Rp1‐D21_constant were not shared with at least one of the Rp1‐D conditions (Figure S8). In contrast, only 110 of 376 up‐regulated DEGs (29%) in Rp1‐D21_48hr were not shared with at least one of the Rp1‐D conditions (Figure S8). This indicated that, as expected, the initial consequences of activating Rp1‐D and Rp1‐D21 were substantially similar.

We examined the 853 DEGs that were shared between at least one Rp1‐D and one Rp1‐D21 condition for enriched GO terms. Seventy‐five statistically enriched GO categories were identified amongst the up‐regulated DEGs (File S6). Among these we identified six categories for further inspection based on their degree of enrichment and biological interest. Enriched GO terms included phenylpropanoid metabolic process (GO:0009698), response to fungus (GO:0009620), cell surface receptor signalling pathway (GO:0007166), defence response (GO:0006952), oxidation‐reduction process (GO:0055114), and transmembrane transport (GO:0055085) (Figure S8b). In total, 198 genes were commonly up‐regulated under all four conditions (File S7). Of these, 108 of the DEGs were distributed among nine enriched GO terms, of which 32% were classified into metabolic process (GO:0008152) and 34% in cellular process (GO:0009987).

## 3 DISCUSSION

The *P. sorghi–*maize interaction has been the subject of previous studies (Hulbert, 1997; Pryor, 1994); however, the regulatory and metabolic pathways associated with the compatible and incompatible defence responses induced during this interaction have not been characterized previously. We generated transcriptome and metabolome profiles of resistant and susceptible near‐isogenic maize lines (H95:Rp1‐D and H95, respectively) infected by *P. sorghi* isolate IN2. The maize *Rp1‐D* gene confers race‐specific resistance against isolates of *P. sorghi*. While the genetics underlying the host range of the pathogen have not been determined, our assumption is that this interaction follows the gene‐for‐gene paradigm elucidated by Flor (1942, 1971) and that *P. sorghi* isolates that are avirulent to maize lines carrying *Rp1‐D* themselves carry a dominant avirulence gene whose product is recognized by Rp1‐D.

Because we were interested in identifying genes induced specifically due to activation of Rp1‐D upon *P. sorghi* infection without confounding effects as a result of the genetic background, it was critical to confirm that the two lines used in the study, H95:Rp1‐D and H95, were truly near‐isogenic, differing for few genes except *Rp1‐D* itself. We observed near‐identical genetic profiles and only 19 DEGs between H95 and H95:Rp1‐D grown under identical conditions prior to infection. By comparison, more than 4,500 DEGs were identified between the commonly used maize lines B73 and Mo17 grown under standard greenhouse conditions (Song et al., 2017). Therefore, it is clear therefore that H95 and H95:Rp1‐D are largely (>c.98%) isogenic and therefore the differences between the H95 and H95:Rp1‐D defence responses are largely due to activation of Rp1‐D.

Genome‐wide transcriptome analyses of the H95 and H95:Rp1‐D lines upon *P. sorghi* infection revealed an initial common response shared between the resistant line H95:Rp1‐D and the susceptible line H95 combined with a defence response specific for the resistant line. Almost all DEGs (c.90%) found in H95 at the earlier time points (12 and 24 hpi) were shared with the H95:Rp1‐D line, indicating that the H95 does not exhibit a specific response and that the common defence response is also triggered within the resistant line (Figure S4). In addition to the DEGs shared with the susceptible line, the resistant line appeared to exhibit an additional response. We reasoned that the molecular mechanisms underlying resistance are most likely to be reflected within this unique set of DEGs.

Targeted metabolomics revealed a common defence response, as well as a response specific to the resistant line. This suggested that resistance to *P. sorghi* is conferred by a prompt ETI response that includes HR cell death as one component. However, several questions remained: What types of genes are differentially regulated during ETI? Which transcriptional interactions are causal for ETI‐induced cell death? Using GO term enrichment analysis and by inferring causal relations within the resistance‐specific set of DEGs, we addressed some of these questions. We constructed a complex GRN (Figure 7) and identified several potential hub TFs, which are obvious candidates for subsequent in planta validation. The WRKY TF family plays crucial roles in the response to biotic stresses in a wide range of plant species (Agostini et al., 2019; Liu et al., 2019; Wei et al., 2012; Yu et al., 2018). Several WRKY TFs were identified upstream in the resistance‐specific GRN at both 12 and 24 hpi, the most significant of these being WRKY53 (Figure 7). *WRKY53* was up‐regulated during the maize response to *C. graminicola*, a hemibiotrophic fungal pathogen and causal agent of anthracnose leaf blight, during the biotrophic phase (24 hpi) (Hoopes et al., 2019) and also in response to infection with *Aspergillus flavus*, the causal agent of aspergillus ear rot (Fountain et al., 2015). Additionally, *ZmWrky53* maps to a region associated with resistance to maize lethal necrosis disease caused by a coinfection of maize chlorotic mottle virus and a potyvirus, and its putative *Arabidopsis* ortholog, *AtWrky33*, is important in the defence response (Birkenbihl et al., 2012; Fountain et al., 2015). These findings together with its prominent place in our derived GRNs suggest that *Wrky53* may be a key player in the maize defence response.

Similarly, we predicted causal regulations between the DEGs common to H95 and H95:Rp1‐D to gain insights into the common response observed within 1 day of infection (Figures S6 and S7). Interestingly, within the common response GRN, several metabolite‐associated genes were predicted to be coregulated by a few TFs at both 12 hpi and 24 hpi. Four of the predicted upstream regulators of metabolite‐associated genes, WRKY73 (*Zm00001d043663*), WRKY34 (*Zm00001d009939*), WRKY68 (*Zm00001d011133*), and bHLH94 (*Zm00001d044272*), were common between the two time points and appear to form part of a network module that co‐regulates these metabolite‐associated genes (see orange boxes in Figures S6 and S7). These TFs would therefore be interesting candidates for further study in relation to the response to infection by *P. sorghi* and other pathogens.

In this study, we observed several genes associated with the PPP and the flavonoid and HCAA pathways that were differentially regulated during the defence response and that the PPP was, overall, one of the most highly enriched GO terms among the DEGs (Figures 3, 4, 5). The 19 DEGs in the PPP and the flavonoid pathway were generally induced in both resistant and susceptible interactions, but in most cases were induced earlier and at higher levels in the resistant H95:Rp1‐D line (Figures 4 and 5). Altogether, these results strongly suggest an important role for the PPP and derived pathways during the interaction between rust‐resistant maize lines and *P. sorghi*. This is also interesting in light of the fact that we have previously identified two PPP enzymes important for lignin biosynthesis, HCT and CCoAOMT, as important regulators of Rp1‐D21 (Wang & Balint‐Kurti, 2016; Wang, He, et al., 2015).

Several metabolites in the PPP and HCAA pathways were strongly induced upon *P. sorghi* infection. The role of phenylpropanoids and lignins during HR has been noted previously (Southerton & Deverall, 1990). Chlorogenic acid, caffeic acid, and ferulic acid levels are increased in both lines, although to a higher extent in H95:Rp1‐D, implying that accumulation of these compounds and subsequent lignin synthesis may be required for the common defence response (Figure 4). Flavonoids are an important subsidiary class of compounds derived from the PPP (Shi & Xie, 2014; Tian et al., 2015; Xu et al., 2015). Rapid accumulation of flavonoids at the site of pathogen infection has been observed in a number of systems, including sorghum (Kawahigashi et al., 2016), barley (Karre et al., 2019), soybean (van Soria‐Guerra et al., 2010; de Mortel et al., 2007), black poplar (Ullah et al., 2017), and apple (Lu et al., 2017). Coumaric acid and its flavonoid derivatives naringenin and luteolin are specifically induced in H95:Rp1‐D and hence might be involved in *P*. *sorghi* resistance. In the resistance‐specific 12 hpi GRN, ZmMYB100 (Zm00001d030644) was predicted to regulate LAC23, an enzyme that is involved in lignin biosynthesis. ZmMYB100 has been shown to bind to the promoters of *Hct6*, *Comt1*, and *4Cl3* in the PPP and induce their transcription, leading to the synthesis of phenylpropanoids and lignins (Yang et al., 2017). This suggests that ZmMYB100 may regulate the downstream gene expression for lignin accumulation in the PPP upon infection with an avirulent *P*. *sorghi* isolate.

We also found several terpenoids, kauralexins and zealexins, to be differentially accumulated upon *P. sorghi* (Figure 5). Terpenoids play a major role in the response to several biotic stresses (Bell et al., 1994; Cheng et al., 2007). Several reports have suggested that kauralexins contribute to disease resistance in maize against several necrotrophic and opportunistic pathogens due to their antifungal properties (Christensen et al., 2018; Meyer et al., 2017; Schmelz et al., 2011, 2014; Veenstra et al., 2019). In this study, although six genes in the terpenoid pathway were substantially up‐regulated in both resistant and susceptible responses upon *P. sorghi* infection all time points, accumulation of kauralexins was mainly specific in H95:Rp1‐D (Figure 5). These results suggest that kauralexins may play a similar role in *Rp1‐D*‐mediated resistance and that there may be some level of differential posttranscriptional regulation of enzyme activity between the resistant and susceptible lines.

Two genes involved in SA biosynthesis, *Calmodulin‐binding protein 60G* (*Zm00001d023843*) and *Systemic acquired resistance deficient 1* (*SARD 1*, *Zm00001d039963*) (Sun et al., 2018), were induced during infection in both H95:Rp1‐D and H95 (File S8). In addition to up‐regulation of SA‐responsive genes, SA itself significantly accumulated in H95:Rp1‐D at 24 hpi (Figure 6). However, 12‐OPDA, JA, and JA‐Ile did not significantly accumulate (Figure 6), and JA levels were actually suppressed in H95:Rp1‐D at 48 hpi. It is possible that, upon *P. sorghi* infection, induction of SA levels suppressed subsequent JA accumulation. Antagonism between JA and SA signalling has been observed in several other systems (e.g., Takahashi et al., 2004; De Vleesschauwer et al., 2013;). These data suggest that SA rather than JA signalling may contribute to HR‐mediated resistance to *P. sorghi*.

Down‐regulated DEGs were significantly enriched in GO term categories associated with the light response, photosynthesis, and sucrose and energy production (Figure 3). Similar observations have been made in other studies of the plant transcriptional defence response (Attaran et al., 2014; Duan et al., 2020; Tzin et al., 2017). The suppression of photosynthesis may be the result of temporary resource allocations to pathogen defence.

We previously examined the spontaneous HR mediated by Rp1‐D21, an autoactive derivative of Rp1‐D. In a series of studies, we identified loci and genes modulating the Rp1‐D21‐mediated response (Olukolu et al., 2014; Wang & Balint‐Kurti, 2016) and characterized environmental conditions, subcellular localization, and inter‐ and intramolecular interactions influencing its activity (Negeri et al., 2013; Wang & Balint‐Kurti, 2015; Wang, Ji, et al., 2015). We suggested that the Rp1‐D21‐mediated response was an exaggerated form of the wild‐type HR mediated by Rp1‐D and thus that the factors important in modulating the Rp1‐D21‐mediated response were probably also important in the HR mediated by Rp1‐D and perhaps for HRs mediated by other NLRs. Here, we show that the transcriptional responses induced by Rp1‐D21 and Rp1‐D are broadly similar. Other studies have shown that the lignin (Wang & Balint‐Kurti, 2016; Wang, He, et al., 2015) and flavonoid biosynthesis (Zhu et al., 2020) pathways are both induced by Rp1‐D21 and are also important for its regulation. The broad similarities between these phenomena provide further justification to extrapolate findings made with Rp1‐D21 to its wild‐type counterpart Rp1‐D.

In conclusion, transcriptomic and metabolomic assays demonstrated the activation of secondary metabolite pathways including phenylpropanoids, flavonoids, terpenoids, and phytoalexins during the defence response in both lines. By almost all criteria, the response was faster and stronger in the resistant response mediated by the R protein Rp1‐D. By constructing GRNs, we also identified a number of TFs that are probably involved in the Rp1‐D‐mediated defence response. This combined approach including transcriptomics, metabolomics, and GRN analysis provides novel insights to understand the maize–*P. sorghi* interaction. The large transcriptomic data set generated here will be a useful resource for further analysis of maize biotic stress responses.

## 4 EXPERIMENTAL PROCEDURES

### 4.1 Plant materials and *P. sorghi* IN2 inoculations

The maize line H95:Rp1‐D is near‐isogenic to the commonly used maize line H95 with the addition that it carries the *Rp1‐D* resistance gene in a homozygous state. Seedlings of H95:Rp1‐D and H95 were grown under a 16 hr/8 hr photoperiod cycle, at day/night temperatures of 26/22 °C and a relative humidity of 60% in growth chambers in the NCSU phytotron. Two‐week‐old plants at the five‐leaf stage were inoculated using spores of *P. sorghi* IN2 and placed in 100% humidity for 16 hr. One hundred microlitres of freshly collected spores from H95 infected by *P. sorghi* IN2 at 7 dpi was mixed with 900 µl of talc and rubbed using a thumb and index finger method onto the fourth and fifth leaves. Inoculated seedlings were placed at 100% humidity in a plastic tent for 16 hr after which they were returned to the original conditions: 16 hr/8 hr photoperiod, day/night temperatures of 26/22 °C, and a relative humidity of 60%. For mock inoculation, H95 was rubbed with talcum powder without the spores but was otherwise treated identically to the inoculated plants.

### 4.2 Random amplified polymorphic DNA analysis

Genomic DNA of samples was isolated using a standard phenol/chloroform extraction method (Allen et al., 2006). RAPD primers used are shown in Table S1. PCRs were performed using 50 ng template DNA. DreamTaq PCR master mix (Thermo Fisher Scientific) was used for PCR (DreamTaq DNA polymerase, 2 × DreamTaq buffer, dATP, dCTP, dGTP, and dTTP, 0.4 mM each, and 4 mM MgCl_2_). The PCR cycle used was 98 °C for 5 min; followed by 45 cycles of 98 °C for 30 s, 35 °C for 30 s, and 68 °C for 1 min; with a final extension of 5 min at 65 °C.

### 4.3 Trypan blue staining

Discs from infected leaves were sampled at 48 hpi and put into 6‐well plates with 0.05% trypan solution (20 mg trypan blue in 10 ml water, 10 ml lactic acid, 10 ml phenol). Samples were placed in a vacuum chamber at 20 mm Hg for 5 min and then released. This step was repeated four times and the plates were then placed in boiling water for 5 min and kept at room temperature overnight. Samples were then placed in 1.95 M KOH solution for 6 hr to remove the chlorophyll and were washed using phosphate‐buffered saline three times. Images were captured using a light microscope.

### 4.4 Experimental design, RNA isolation and sequencing

For each experimental replicate, tissue from six individual maize plants was pooled for each condition. Conditions were classified by treatment/genotype (mock_H95, infected_H95, and infected_H95:Rp1D) and time point (0, 12, 24, and 120 hpi) for a total of 12 conditions with six plants per condition, meaning each replication consisted of 72 plants in total. Within these 72 plants, the inoculated and mock‐inoculated plants were maintained in separate sides of the same growth chamber to minimize cross‐contamination. Within each of these sets of plants, inoculated or mock‐inoculated, the plants were randomized with respect to genotype and time point. Three independent replicates of this experiment were performed over three consecutive weeks. Thus, in total, RNA from 216 plants was isolated and analysed for this study.

Samples were frozen in liquid nitrogen and ground to a fine powder using a mortar and pestle. Total RNA was extracted using TRIzol (Invitrogen) according to the manufacturer's instructions. Libraries were constructed using the Illumina TruSeq stranded mRNA kit following the manufacturer's instructions. The starting quantity of total RNA was adjusted to 1.2 µg per sample. Deep sequencing was performed using an Illumina NovaSeq 6000 for 150 paired‐end sequencing.

### 4.5 Transcriptome data analysis

The raw sequence data supporting the results of this article are available in the Short Read Archive (accession number PRJNA634448; https://www.ncbi.nlm.nih.gov/sra/PRJNA634448). The raw sequence reads were mapped to v4 of the B73 cDNA reference database (Zm00001d.2; https://www.maizegdb.org/) using the BBMap aligner (https://sourceforge.net/projects/bbmap/). Using the bbmap.sh command, we obtained reads per kilobase of transcript per million mapped reads (RPKM) values for each library. PCA was performed in R v. 4.0.2 using the prcomp package. The obtained RPKM values from each condition were further analysed by DEBrowser for DEG identification (Kucukural et al., 2019). DEGs with *p* values of less than 0.05 and log_2_‐converted fold changes (FCs) of more than log_2_(FC) ≥ 2 or ≤ −2 were identified. DEGs with known *Zea mays* orthologs were then mapped to the GO database using PANTHER v. 14.1 (http://geneontology.org/). GO enrichment analysis in PANTHER was performed for down‐ and up‐regulated DEGs separately. Next, all enriched GO terms (FDR < .05 and fold enrichment > 1.5) were pooled and summarized with REVIGO (Supek et al., 2011) to remove redundant GO terms. GO terms with a dispensability larger than 0.5 and found in at least two DEG sets were visualized using RStudio. MapMan software (Thimm et al., 2004) was also used for biochemical pathway analysis. GO enrichment analysis was performed using a significance level of FDR < .05.

### 4.6 RT‐qPCR

First‐strand cDNA was synthesized using an oligo (dT) primer and 3 μg of total RNA with Superscript II reverse transcriptase (Invitrogen), followed by qPCR with gene‐specific primers (Table S2). RT‐qPCR was performed on the StepOnePlus Real‐Time PCR System (Thermo Fisher Scientific) using SsoAdvanced Universal SYBR Green (Bio‐Rad). All calculations and statistical analyses were performed as described by the manufacturer. *Actin* was used as the endogenous control.

### 4.7 Gene regulatory network analysis

After identifying the DEGs between the mock and the susceptible line and between the mock and the resistant line, we selected the DEGs unique to the defence response in the resistant line at 12 and 24 hpi to explore regulations that potentially confer resistance. We inferred two GRNs with a regression tree and random forest algorithm. Specifically, we leveraged the network inference software RTP‐STAR within the GUI TuxNet (Spurney et al., 2020). For the algorithm, we used the biological replicates of the 12 and 24 hpi expression data of the mock and H95:Rp1‐D‐infected line to predict causal relations between the unique DEGs. We performed 10 iterations and only included the regulations found in 66% of the iterations. The direction of the inferred regulations (activation, repression, or undetermined) was determined with the complete time‐course data set of H95:Rp1‐D.

### 4.8 Targeted metabolomics

Ground and frozen tissue (50 mg) in 1.5‐ml FastPrep tubes was carefully thawed to 4 °C and then transferred to ice. An internal standard mix of 12 μl containing caffeine, D6‐abscisic acid, D5‐jasmonic acid, D5‐cinnamic acid, D5‐indole‐3‐acetic acid, [^13^C]‐α‐linolenic acid, and nicotine at 8.33 μg/ml was added and plant metabolite extractions were performed as described by S. A. Christensen et al. (personal communication). Ultra‐high‐performance liquid chromatography–high‐resolution mass spectrometry (UHPLC‐HRMS) was carried out on a Q Exactive mass spectrometer coupled to a Vanquish LC System (Thermo Fisher Scientific) by reverse phase gradient elution using an ACE Excel 2 C18‐PFP column (2.1 μm, 100 mm) in full scan positive (injection volume 2 μl) and negative (injection volume 4 μl) ion modes, and the data were acquired, processed, normalized, and filtered and metabolites were identified using MZmine 2 (Pluskal et al., 2010) and MetaboAnalyst 4.0 (Chong et al., 2018) software.

## Supporting information


**FIGURE S1** Random amplified polymorphic DNA (RAPD) analysis of B73, Mo17, H95, and H95:Rp1‐D maize lines. “Vial” numbers refer to primers described in Table S1

**FIGURE S2** Principal component analysis (PCA) of the RNA‐Seq samples. RPKM values of all the replicates of the mock (M), H95 (S), and H95:Rp1‐D (R) samples were used to perform a PCA. M.0, M.12, M.24, and M.120 represent mock‐treated H95 at 0, 12, 24, and 120 hr postinoculation (hpi). S.0, S.12, S.24, and S.120 represent RNA‐Seq from *Puccinia sorghi*‐infected H95 at 0, 12, 24, and 120 hpi. R.0, R.12, R.24, and R.120 represent RNA‐Seq from *P. sorghi*‐infected H95:Rp1‐D at 0, 12, 24, and 120 hpi. In the PCA plot, each dot represents an RNA‐Seq sample. The samples are plotted in two dimensions using their projections onto the first two principal components

**FIGURE S3** *Puccinia sorghi* infection‐induced defence gene expression in maize (*n* = 3, average ± SEM). Graphs show quantitative reverse transcription PCR (RT‐qPCR) and RNA‐Seq fold changes in transcript levels of genes encoding pathogen defence proteins, including (a) *ZmCHI* (Zm00001d044683), (b) *ZmAOS4* (Zm00001d0341840), (c) *ZmCalmodulin* (Zm00001d023843), (d) *ZmPR5* (Zm00001d031158), (e) *ZmLOX3* (Zm00001d033623), and (f) *ZmWRKY104* (Zm00001d020495). *ZmActin* (Zm00001d010159) was used as internal control. All tissues were sampled at 0, 12, 24, and 120 hr postinoculation (hpi). Lines and histograms represent the relative expression levels (fold change) as assessed by RT‐qPCR (left *y* axis) and RNA‐Seq (right *y* axis), respectively. ***p < .001 (*t* test). N.B. For RNA‐Seq data if the differential expression was not statistically significant for a specific time point, then the fold change was recorded as “1.”

**FIGURE S4** Venn diagram indicating the shared distribution of differentially expressed genes (DEGs) at each time point in H95 and H95:Rp1‐D

**FIGURE S5** The distribution of differentially expressed genes (DEGs) from *Puccinia sorghi*‐infected H95:Rp1‐D and H95 plants at 24 and 120 hr postinoculation (hpi) among various cellular processes, visualized by MapMan. The intensity of the colour indicates the level of differential expression. Scale bar displays log_2_(fold change) values. Red and blue colours represent up‐ and down‐regulation, respectively

**FIGURE S6** Inferred gene regulatory network (GRN) underlying the common response at 12 hr postinoculation (hpi). Causal regulatory interactions were inferred between the differentially expressed genes (DEGs) shared between the H95:Rp1‐D and H95 lines at 12 hpi. Transcription factors (TFs) and the metabolic enzyme‐associated genes are shown with red and green borders, respectively. Each gene is coloured according to induction level. The orange square indicates a high number of metabolite‐associated genes that are coregulated within the network

**FIGURE S7** Inferred gene regulatory network (GRN) underlying the common response at 24 hr postinoculation (hpi). Causal regulatory interactions were inferred between the differentially expressed genes (DEGs) shared between the H95:Rp1‐D and H95 lines at 24 hpi. TFs and the metabolic enzyme‐associated genes are shown with red and green borders, respectively. Each gene is coloured according to induction level. The orange square indicates a high number of metabolite‐associated genes that are coregulated within the network

**FIGURE S8** Distribution of up‐regulated differentially expressed genes (DEGs) among H95:Rp1‐D infected with *Puccinia sorghi* at 24 hr postinoculation (hpi) and various treatments of B73 × H95:Rp1‐D21 plants as explained in the text. (a) Venn diagram indicating the distribution of up‐regulated DEGs among time points. (b) Selected enriched GO terms among the 853 genes included in labelled red boxes in Figure S8a

**TABLE S1** Primers used for random amplified polymorphic DNA (RAPD) analysis

**TABLE S2** Primers used for quantitative reverse transcription PCR

**FILE S1** The details of the 19 differentially expressed genes (DEGs) between H95 and H95:Rp1‐D prior to infection

**FILE S2** Read mapping summary for H95 and H95:Rp1‐D. The sheet named “H95 to B73” shows reads mapped to v4 of B73. The sheet named “H95 to Puccinia” shows reads mapped to *Puccinia sorghi* RO10H11247

**FILE S3** Significantly enriched or under‐represented GO terms among differentially expressed genes (DEGs) divided into six worksheets by each of the three time points and the two genotypes as indicated by the worksheet titles. In each worksheet, Column A shows the GO term description and the GO accession in biological process (BP). Column B shows the number of annotated genes in the *Zea mays* genome that are associated with this particular category. Column C shows the number DEGs that are annotated in this category. Column D shows the number of DEGs we would expect in this category with no enrichment or under‐representation. Column E contains either a plus sign (+) or a minus sign (−). A plus sign indicates over‐representation and a negative sign indicates under‐representation. Column F indicates the fold enrichment or depletion compared to expected. Column G shows the *p* value determined by Fisher’s exact test. Column H contains the false discovery rate as calculated by the Benjamini–Hochberg procedure. A critical value of 0.05 was used to filter results. The worksheet entitled “Unique enriched GO terms” simply lists the 238 unique GO terms that were enriched in at least one of the conditions

**FILE S4** Output of the network inference algorithm and results of the regression tree analysis performed with RTP‐STAR in TuxNet software. The first four sheets contain the inferred causal relations between a transcription factor (TF) and downstream target genes of the four inferred networks: 1. H95Rp1‐D_12hpi (resistance‐specific network at 12 hr postinoculation [hpi], Figure 7), 2. H95Rp1‐D_24hpi (resistance‐specific network at 24 hpi, Figure 7), 3. Common_12hpi (general defence response network at 12 hpi, Figure S6), and 4. Common_24hpi (general defence response network at 24 hpi, Figure S7). The following columns are present: source (i.e., the upstream transcriptional regulator), interaction (i.e., activation, inhibition, or regulation in case of an undetermined interaction), the target (i.e., the downstream gene), and the width of the interaction (i.e., a confidence score). The fifth sheet (“Overlap_ChIP”) details the source nodes (TFs) that were found in a publicly available network built with ChIP data (Tu et al., 2020), the outdegree (i.e., the number of outgoing edges and thus downstream targets), and the percentage of downstream targets also identified in the aforementioned network

**FILE S5** Output of a GO enrichment analysis with the BiNGO app in Cytoscape. The BiNGO analysis was performed on the non‐TF nodes of the 12 hr postinoculation (hpi) and 24 hpi networks. X = total number of genes in the tested gene set, N = total number of genes in the maize genome, x = number of genes from the tested gene set that are associated to the GO ID, n = number of genes from the maize genome that are associated with the GO ID

**FILE S6** Significant enriched or under‐represented GO terms for among the 853 genes included in labelled red boxes in Figure S8a. Data in each column are as described in File S3

**FILE S7** List of 198 genes commonly up‐regulated between H95:Rp1‐D infected with *Puccinia sorghi* IN2 and B73 × H95:Rp1‐D21 under all conditions as shown in Figure S8a

**FILE S8** Details of differentially expressed genes (DEGs) from selected GO terms induced in H95:Rp1‐D and H95 by *Puccinia sorghi* IN2 infection. Log_2_(fold induction) levels are indicated. Asterisks (*) indicate the genes to validate the transcript expression levels for quantitative reverse transcription PCR

## Data Availability

The data that support the findings will be available in the Short Read Archive (https://www.ncbi.nlm.nih.gov/sra/PRJNA634448) under accession number PRJNA634448 following an embargo from the date of publication to allow for commercialization of research findings.
