# Supplementary material for: Analysis of the transcriptomic, metabolomic, and gene regulatory responses to Puccinia sorghi in maize
Source: Mol Plant Pathol. 2021 Feb 28;22(4):465–79. doi: 10.1111/mpp.13040 (PMC7938627; doi:10.1111/mpp.13040)
Supplement: Supplementary file 1 — FIGURE S1 Random amplified polymorphic DNA (RAPD) analysis of B73, Mo17, H95, and H95:Rp1‐D maize lines. “Vial” numbers refer to primers described in Table S1 [file MPP-22-465-s008.pdf]

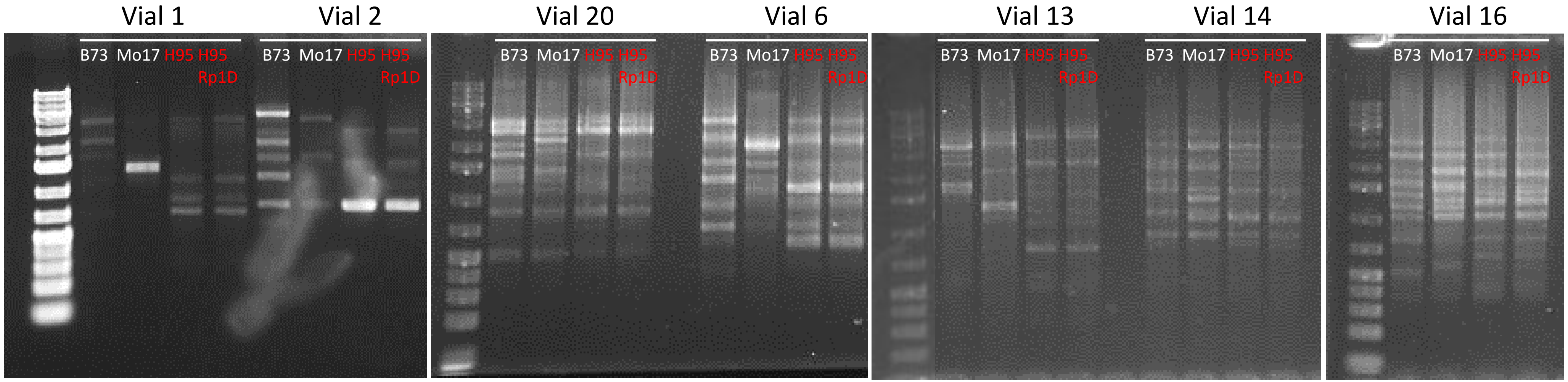

Supplementary Figure 1. RAPD analysis of B73, Mo17, H95 and H95:Rp1-D maize lines. “Vial” numbers refer to primers described in Table S1.
