# Supplementary material for: Analysis of the transcriptomic, metabolomic, and gene regulatory responses to Puccinia sorghi in maize
Source: Mol Plant Pathol. 2021 Feb 28;22(4):465–79. doi: 10.1111/mpp.13040 (PMC7938627; doi:10.1111/mpp.13040)
Supplement: Supplementary file 2 — FIGURE S2 Principal component analysis (PCA) of the RNA‐Seq samples. RPKM values of all the replicates of the mock (M), H95 (S), and H95:Rp1‐D (R) samples were used to perform a PCA. M.0, M.12, M.24, and M.120 represent mock‐treated H95 at 0, 12, 24, and 120 hr postinoculation (hpi). S.0, S.12, S.24, and S.120 represent RNA‐Seq from Puccinia sorghi‐infected H95 at 0, 12, 24, and 120 hpi. R.0, R.12, R.24, and R.120 represent RNA‐Seq from P. sorghi‐infected H95:Rp1‐D at 0, 12, 24, and 120 hpi. In the PCA plot, each dot represents an RNA‐Seq sample. The samples are plotted in two dimensions using their projections onto the first two principal components [file MPP-22-465-s010.pdf]

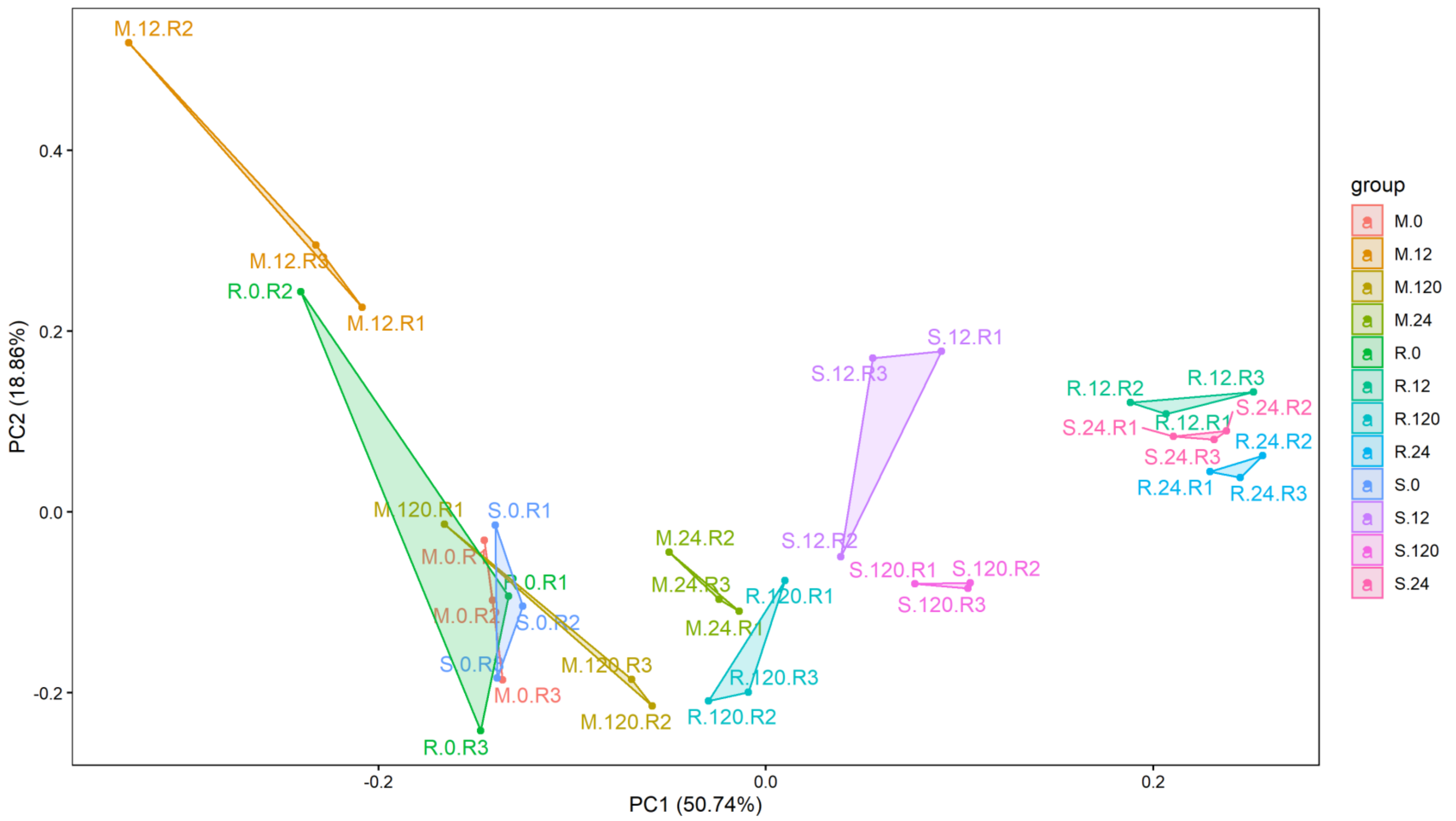

Supplementary Figure 2. Principal Component analysis (PCA) of the RNAseq samples. RPKM values of all the replicates of the Mock, H95, and H95:Rp1-D samples were used to perform a PCA analysis. In the PCA plot, each dot represents an RNAseq sample. The samples are plotted in two dimensions using their projections onto the first two principal components.
