# Supplementary material for: Analysis of the transcriptomic, metabolomic, and gene regulatory responses to Puccinia sorghi in maize
Source: Mol Plant Pathol. 2021 Feb 28;22(4):465–79. doi: 10.1111/mpp.13040 (PMC7938627; doi:10.1111/mpp.13040)
Supplement: Supplementary file 3 — FIGURE S3 Puccinia sorghi infection‐induced defence gene expression in maize (n = 3, average ± SEM). Graphs show quantitative reverse transcription PCR (RT‐qPCR) and RNA‐Seq fold changes in transcript levels of genes encoding pathogen defence proteins, including (a) ZmCHI (Zm00001d044683), (b) ZmAOS4 (Zm00001d0341840), (c) ZmCalmodulin (Zm00001d023843), (d) ZmPR5 (Zm00001d031158), (e) ZmLOX3 (Zm00001d033623), and (f) ZmWRKY104 (Zm00001d020495). ZmActin (Zm00001d010159) was used as internal control. All tissues were sampled at 0, 12, 24, and 120 hr postinoculation (hpi). Lines and histograms represent the relative expression levels (fold change) as assessed by RT‐qPCR (left y axis) and RNA‐Seq (right y axis), respectively. ***p < .001 (t test). N.B. For RNA‐Seq data if the differential expression was not statistically significant for a specific time point, then the fold change was recorded as “1.” [file MPP-22-465-s006.pdf]

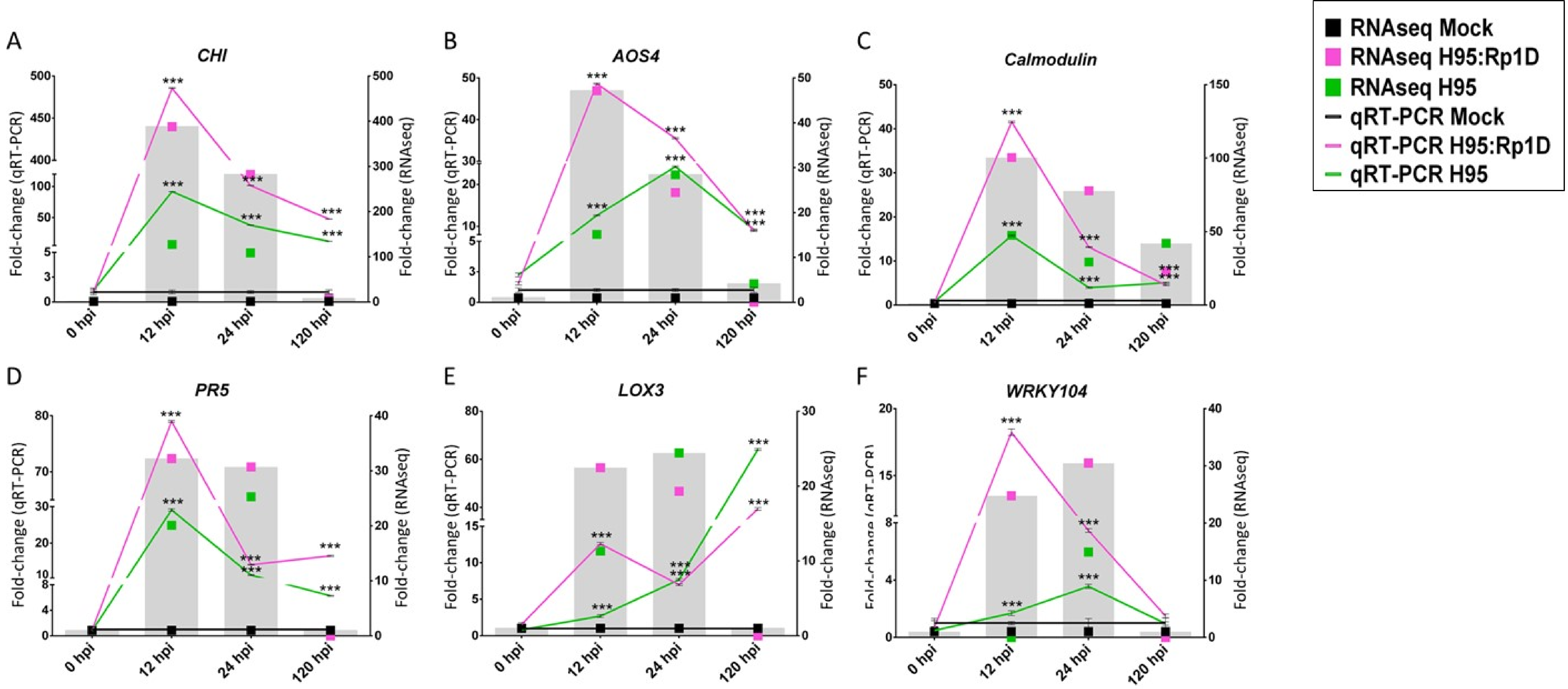

Supplementary Figure 3. *P. sorghi* infection-induced defense gene expression in maize. Average (n = 3, ± SEM). Graphs show qRT-PCR and RNAseq fold change in transcript levels of genes encoding pathogen defense proteins including **A**. *ZmCHI* (Zm00001d044683), **B.** *ZmAOS4* (Zm00001d0341840), **C.** *ZmCalmodulin* (Zm00001d023843), **D.** *ZmPR5* (Zm00001d031158), **E.** *ZmLOX3* (Zm00001d033623), **F.** *ZmWRKY104* (Zm00001d020495). *ZmActin* (Zm00001d010159) was used as internal control. All tissues were sampled at 0, 12, 24, and 120 hpi. Lines and histograms represent the relative expression levels (fold change) as assessed by qRT-PCR (left y-axis) and RNAseq (right y-axis), respectively. *** $p < 0.001$ (t-test). NB for RNAseq data if the differential expression was not statistically significant for a specific timepoint then the fold change was recorded as "1".
