# Supplementary material for: Analysis of the transcriptomic, metabolomic, and gene regulatory responses to Puccinia sorghi in maize
Source: Mol Plant Pathol. 2021 Feb 28;22(4):465–79. doi: 10.1111/mpp.13040 (PMC7938627; doi:10.1111/mpp.13040)
Supplement: Supplementary file 4 — FIGURE S4 Venn diagram indicating the shared distribution of differentially expressed genes (DEGs) at each time point in H95 and H95:Rp1‐D [file MPP-22-465-s013.pdf]

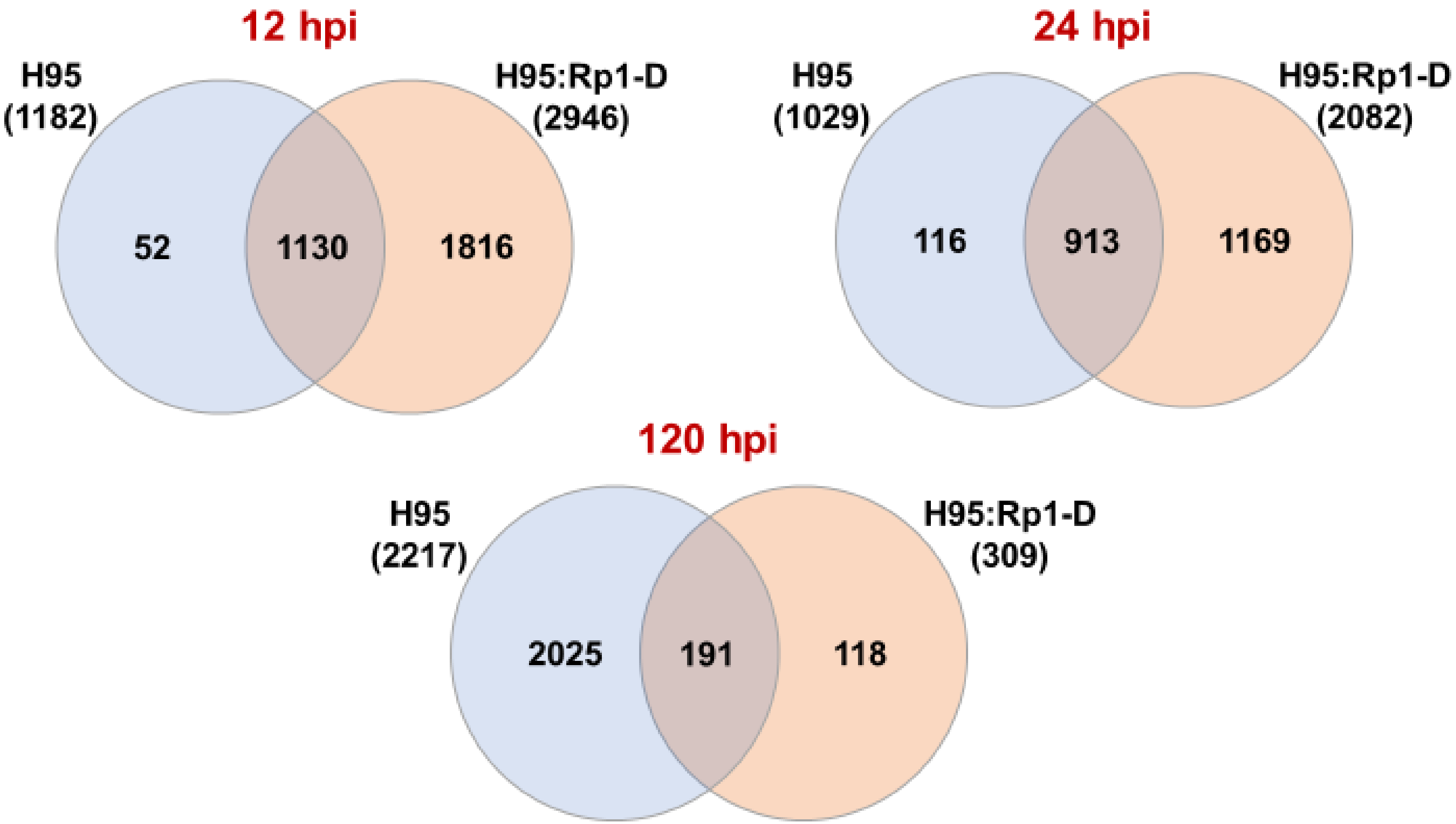

Supplementary Figure 4. Venn diagram indicating the shared distribution of DEGs at each time point in H95 and H95:Rp1-D
