# Supplementary material for: Analysis of the transcriptomic, metabolomic, and gene regulatory responses to Puccinia sorghi in maize
Source: Mol Plant Pathol. 2021 Feb 28;22(4):465–79. doi: 10.1111/mpp.13040 (PMC7938627; doi:10.1111/mpp.13040)
Supplement: Supplementary file 5 — FIGURE S5 The distribution of differentially expressed genes (DEGs) from Puccinia sorghi‐infected H95:Rp1‐D and H95 plants at 24 and 120 hr postinoculation (hpi) among various cellular processes, visualized by MapMan. The intensity of the colour indicates the level of differential expression. Scale bar displays log2(fold change) values. Red and blue colours represent up‐ and down‐regulation, respectively [file MPP-22-465-s003.pdf]

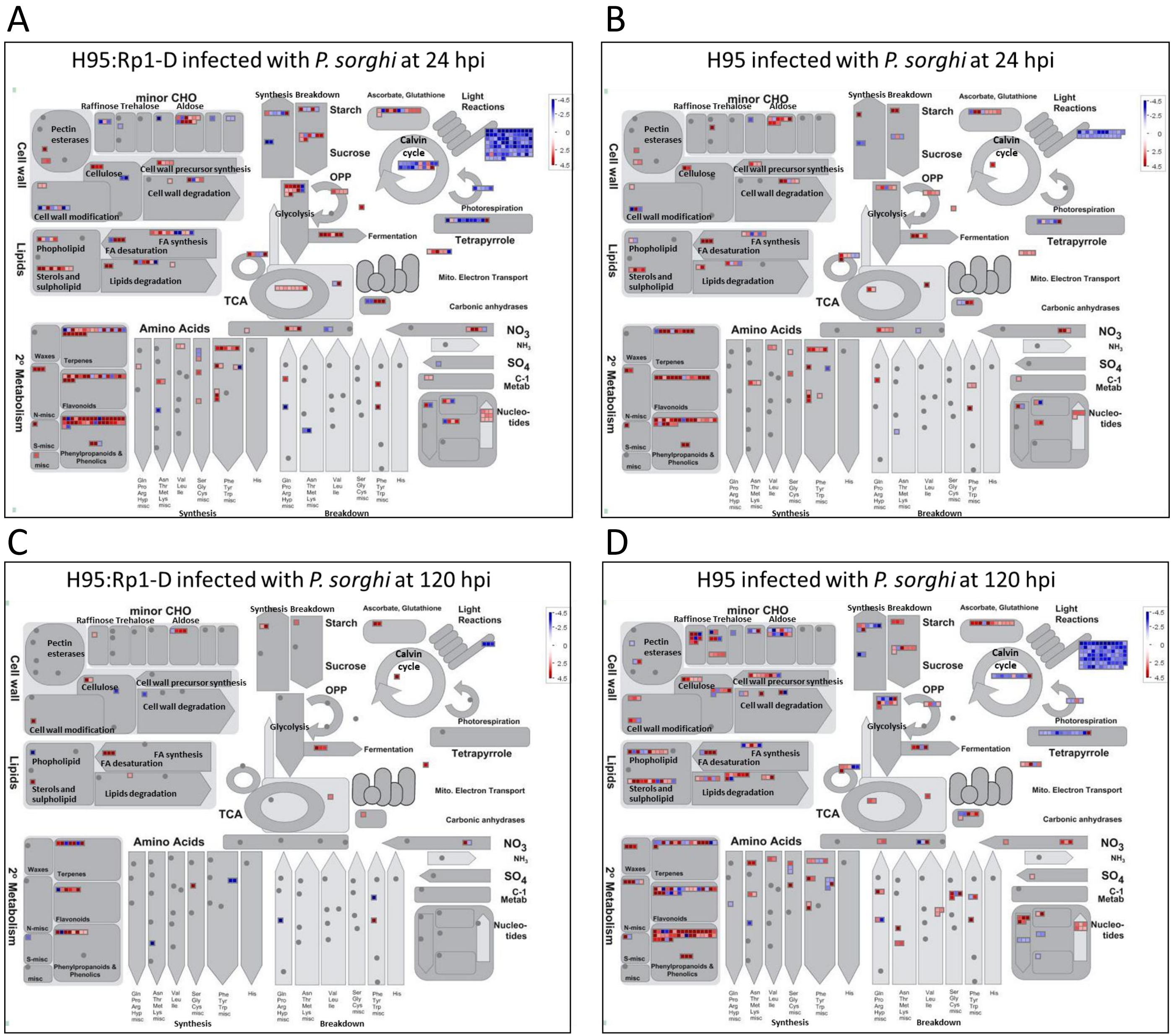

Supplementary Figure 5. The distribution of DEGs from P. sorghi infected H95:Rp1-D and H95 at 24 and 120 hpi among various cellular processes, visualized by MapMan. The intensity of the color indicates the level of differential expression. Scale bar displays Log2-fold-changes. Red or blue colors represent up- and down-regulation, respectively.
