# Supplementary material for: Analysis of the transcriptomic, metabolomic, and gene regulatory responses to Puccinia sorghi in maize
Source: Mol Plant Pathol. 2021 Feb 28;22(4):465–79. doi: 10.1111/mpp.13040 (PMC7938627; doi:10.1111/mpp.13040)
Supplement: Supplementary file 6 — FIGURE S6 Inferred gene regulatory network (GRN) underlying the common response at 12 hr postinoculation (hpi). Causal regulatory interactions were inferred between the differentially expressed genes (DEGs) shared between the H95:Rp1‐D and H95 lines at 12 hpi. Transcription factors (TFs) and the metabolic enzyme‐associated genes are shown with red and green borders, respectively. Each gene is coloured according to induction level. The orange square indicates a high number of metabolite‐associated genes that are coregulated within the network [file MPP-22-465-s016.pdf]

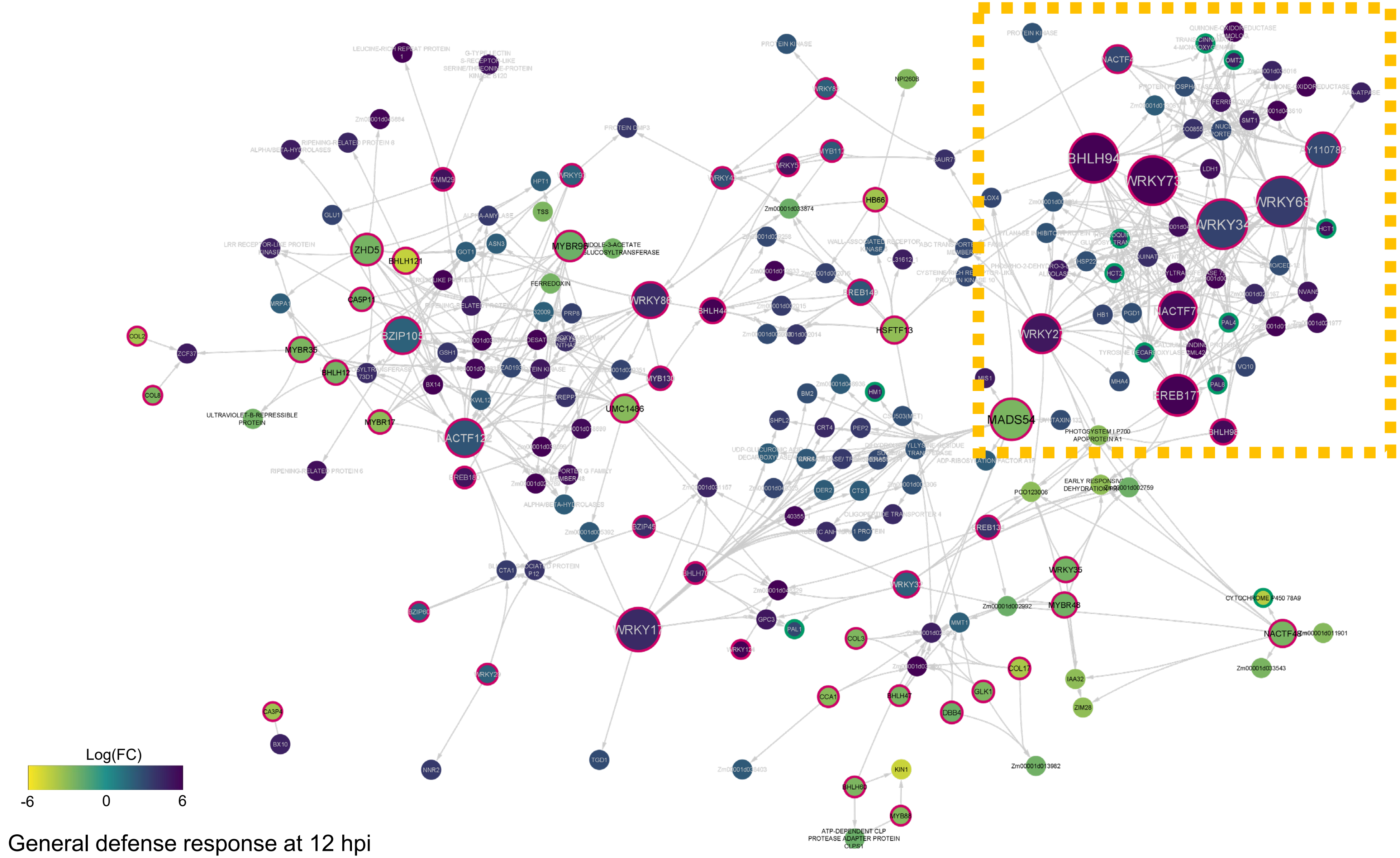

Supplementary Figure 6. Inferred gene regulatory network (GRN) underlying the common response at 12 hpi. Causal regulatory interactions were inferred between the DEGs shared between the H95:Rp1-D and H95 lines at 12 hpi. TFs and the metabolic enzyme-associated genes are shown with red and green borders, respectively. Each gene is colored according to induction level. Orange square indicates a high number of metabolite-associated genes that are co-regulated within the network.
