# Supplementary material for: Analysis of the transcriptomic, metabolomic, and gene regulatory responses to Puccinia sorghi in maize
Source: Mol Plant Pathol. 2021 Feb 28;22(4):465–79. doi: 10.1111/mpp.13040 (PMC7938627; doi:10.1111/mpp.13040)
Supplement: Supplementary file 8 — FIGURE S8 Distribution of up‐regulated differentially expressed genes (DEGs) among H95:Rp1‐D infected with Puccinia sorghi at 24 hr postinoculation (hpi) and various treatments of B73 × H95:Rp1‐D21 plants as explained in the text. (a) Venn diagram indicating the distribution of up‐regulated DEGs among time points. (b) Selected enriched GO terms among the 853 genes included in labelled red boxes in Figure S8a [file MPP-22-465-s001.pdf]

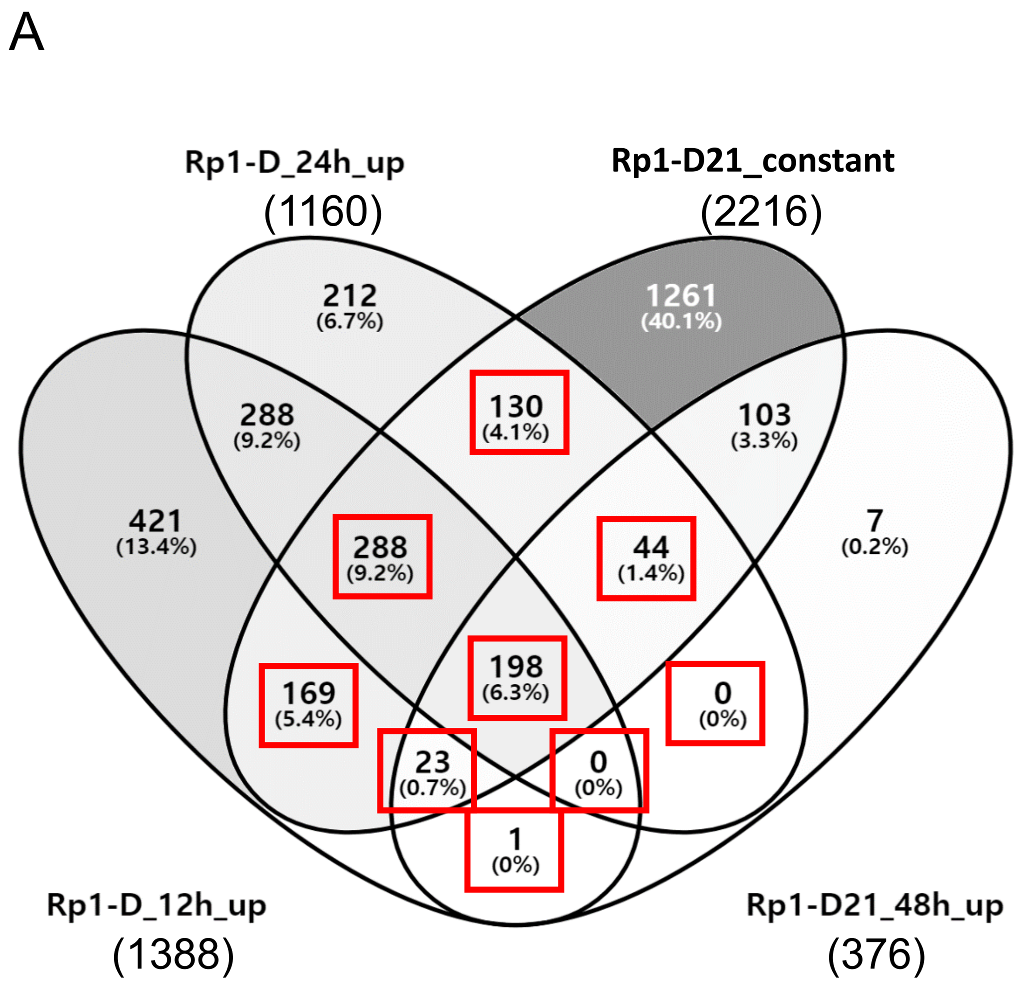

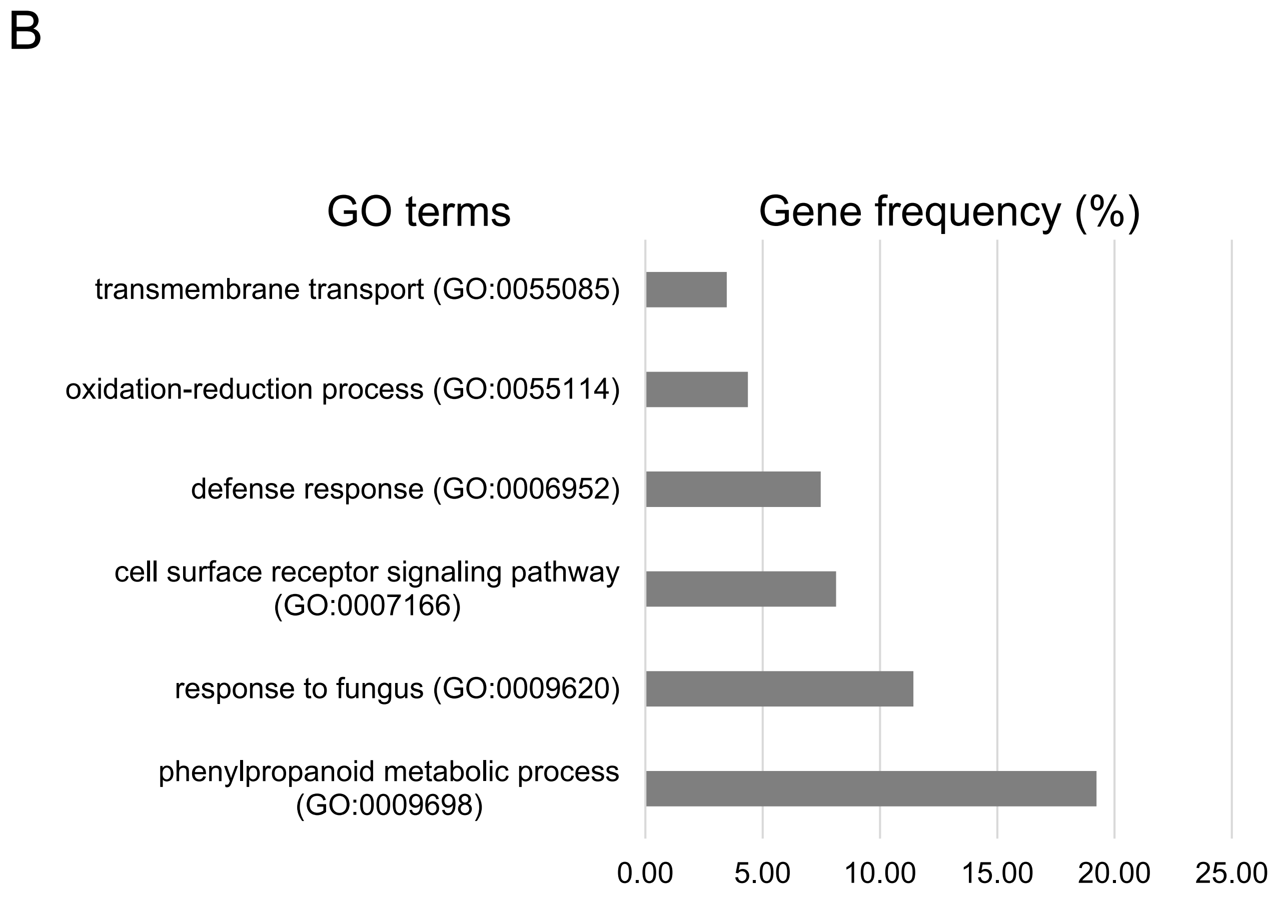

Supplementary Figure 8. Distribution of up-regulated DEGs among H95:Rp1-D infected with *P. sorghi* at 24 hpi and various treatments of B73 x H95:Rp1-D21 plants as explained in the text. **A.** Venn diagram indicating the distribution of up-regulated DEGs among timepoints. **B.** Selected enriched GO terms among the 853 genes included in labeled red boxes in Fig S8A.
