# Supplementary material for: Analysis of the transcriptomic, metabolomic, and gene regulatory responses to Puccinia sorghi in maize
Source: Mol Plant Pathol. 2021 Feb 28;22(4):465–79. doi: 10.1111/mpp.13040 (PMC7938627; doi:10.1111/mpp.13040)
Supplement: Supplementary file 9 — TABLE S1 Primers used for random amplified polymorphic DNA (RAPD) analysis [file MPP-22-465-s015.docx]

| **Table S1.** The list of primers used for RAPD analysis | |
| --- | --- |
| Primer | Sequence (5' to 3') |
| vial1 | ACCTAGGGGA |
| vial2 | TCGCAGGTTC |
| vial6 | TTTGGGCCCC |
| vial13 | CTACGATGCC |
| vial14 | GGTTCTGCTC |
| vial16 | TTACCCCGCT |
| vial20 | TGTCCTAGCC |
