# Supplementary material for: Analysis of the transcriptomic, metabolomic, and gene regulatory responses to Puccinia sorghi in maize
Source: Mol Plant Pathol. 2021 Feb 28;22(4):465–79. doi: 10.1111/mpp.13040 (PMC7938627; doi:10.1111/mpp.13040)
Supplement: Supplementary file 10 — TABLE S2 Primers used for quantitative reverse transcription PCR [file MPP-22-465-s014.docx]

**Table S2**. The list of primers used for RT-qPCR

| Gene ID | Name | F (5' to 3') | R (5' to 3') | Size (bp) |
| --- | --- | --- | --- | --- |
| Zm00001d023843 | ZmCalmodulin | CTTGTACCGATACCATGAGC | CTACTAGCTCCTTTACGTGC | 200 |
| Zm00001d020495 | ZmWRKY104 | ACAACCCGTATCCGAGATC | GTCGCCACAAGCATCAGATT | 106 |
| Zm00001d044683 | ZmCHI | CTGCTCGCCGTCGCCGCGTG | CACAACGACAATCTGCACAAC | 183 |
| Zm00001d010159 | ZmActin | GGTTTCGCTGGTGATGATGC | CAATGCCATGCTCAATCGGG | 169 |
| Zm00001d034184 | ZmAOS4 | TCTATGTAAAATGGCCTGGGA | ATAATTTGCGAGCCCATCCG | 100 |
| Zm00001d033623 | ZmLOX3 | TACCACTACCACCCCAGGAGT | AGCACTGCGAAACGACTAGAA | 233 |
| Zm00001d031158 | ZmPR5 | TCTACGACATCTCGGTCATC | GACTTGGTAGTTGCTGTTGC | 161 |
